# Effects of Soy Protein Isolate on Fragile X Phenotypes in Mice

**DOI:** 10.3390/nu16020284

**Published:** 2024-01-18

**Authors:** Pamela R. Westmark, Greg Lyon, Alejandra Gutierrez, Brynne Boeck, Olivia Van Hammond, Nathan Ripp, Nicole Arianne Pagan-Torres, James Brower, Patrice K. Held, Cameron Scarlett, Cara J. Westmark

**Affiliations:** 1 Department of Neurology, University of Wisconsin, Madison, WI 53706, USA; prwestmark@wisc.edu; 2 Undergraduate Research Scholars Program, University of Wisconsin, Madison, WI 53706, USA; gregorylyon6@gmail.com (G.L.); ovanhammond@wisc.edu (O.V.H.); 3 Molecular Environmental Toxicology Master’s Program, University of Wisconsin, Madison, WI 53706, USA; gutierrez9@wisc.edu; 4 Neurology Undergraduate Research, University of Wisconsin, Madison, WI 53706, USA; bboeck@wisc.edu (B.B.); njripp2@wisc.edu (N.R.); 5 Molecular Environmental Toxicology Summer Research Opportunities Program, University of Wisconsin, Madison, WI 53706, USA; nicole.pagantorres@bsd.uchicago.edu; 6 Wisconsin State Laboratory of Hygiene, University of Wisconsin, Madison, WI 53706, USA; james.brower@slh.wisc.edu (J.B.); patrice.k.held@oha.oregon.gov (P.K.H.); 7 School of Pharmacy, University of Wisconsin, Madison, WI 53706, USA; cameron.scarlett@wisc.edu; 8 Department of Neurology and Molecular Environmental Toxicology Center, University of Wisconsin, Madison, WI 53706, USA

**Keywords:** fragile X syndrome, FMRP, obesity, soy, phytoestrogens, sex-specific differences, actigraphy

## Abstract

Obesity is a pediatric epidemic that is more prevalent in children with developmental disabilities. We hypothesize that soy protein-based diets increase weight gain and alter neurobehavioral outcomes. Our objective herein was to test matched casein- and soy protein-based purified ingredient diets in a mouse model of fragile X syndrome, *Fmr1^KO^* mice. The experimental methods included assessment of growth; 24-7 activity levels; motor coordination; learning and memory; blood-based amino acid, phytoestrogen and glucose levels; and organ weights. The primary outcome measure was body weight. We find increased body weight in male *Fmr1^KO^* from postnatal day 6 (P6) to P224, male wild type (WT) from P32–P39, female *Fmr1^KO^* from P6–P18 and P168–P224, and female *Fmr1^HET^* from P9–P18 as a function of soy. Activity at the beginning of the light and dark cycles increased in female *Fmr1^HET^* and *Fmr1^KO^* mice fed soy. We did not find significant differences in rotarod or passive avoidance behavior as a function of genotype or diet. Several blood-based amino acids and phytoestrogens were significantly altered in response to soy. Liver weight was increased in WT and adipose tissue in *Fmr1^KO^* mice fed soy. Activity levels at the beginning of the light cycle and testes weight were greater in *Fmr1^KO^* versus WT males irrespective of diet. DEXA analysis at 8-months-old indicated increased fat mass and total body area in *Fmr1^KO^* females and lean mass and bone mineral density in *Fmr1^KO^* males fed soy. Overall, dietary consumption of soy protein isolate by C57BL/6J mice caused increased growth, which could be attributed to increased lean mass in males and fat mass in females. There were sex-specific differences with more pronounced effects in *Fmr1^KO^* versus WT and in males versus females.

## 1. Introduction

Fragile X syndrome (FXS) is the leading known genetic cause of autism and the most common form of inherited intellectual disability. This X-chromosome-linked disorder is typically caused by a trinucleotide repeat expansion mutation in the 5′-UTR of the *FMR1* gene that causes transcriptional silencing of the promoter and loss of expression of the fragile X messenger ribonucleoprotein (FMRP) [1,2]. FMRP is an RNA-binding protein with diverse functions including translational regulation and activity-dependent synapse development [3]. Clinical manifestations of FXS include highly variable intellectual disability (overall IQ < 70), autistic-like behaviors, seizures, macrocephaly, and macroorchidism [4]. Children with FXS also exhibit accelerated prepubescent growth, diminution of the normal pubertal growth spurt, and increased incidence of obesity compared to typically developing same-aged peers [5,6,7].

The *FMR1* gene was discovered in 1991, the beginning of the “Decade of the Brain”. Considering the severe intellectual disability in FXS in conjunction with funding initiatives to understand the role of FMRP in brain function, preclinical and clinical research for the disorder has primarily focused on the brain. There is a dearth of research on the role of FMRP in the periphery and in metabolism. While diet is used to treat other disorders such as epilepsy and phenylketonuria (PKU), the role of diet in FXS is understudied. A PubMed search of the term “fragile X” returned over 9000 entries whereas the search terms “fragile X” AND “diet” returned only 39 papers. These publications include research on development and obesity [8,9,10,11], fatty acids [12,13,14,15,16,17,18,19,20,21], carbohydrate [22], proteins [23,24,25,26], dehydroepiandrosterone (DHEA) [27], folate [28,29,30,31], ethanol [32], lithium [33], exposome factors including bisphenol A (BPA) and polychlorinated biphenyls (PCB) [34,35,36,37,38], and the gut microbiome [21,39,40].

In the United States, we have concurrent childhood autism and obesity epidemics with autism prevalence at 3.29% [41] and pediatric obesity at 17% [42]. Developmental disabilities and obesity are highly comorbid, i.e., 22% of children with an autism spectrum disorder have a body mass index (BMI) in the obese range [43,44]. There are disturbances in early infant growth in FXS with increased birth weight and average linear growth in boys [45]. About 10% of individuals with FXS have the Prader–Willi phenotype characterized by unusual obesity, lack of satiation and hyperphagia [46].

There is poor understanding of how infant nutrition interacts with genetics to affect weight gain and neurological development. Infant formula intolerance is 12% [47] suggesting that a high percentage of babies switch formulas. The leading alternative to cow milk-based infant formula is soy-based. Thus, it would be useful to identify growth, behavioral and molecular outcome measures correlated with the consumption of soy protein, particularly in regards to neurodevelopmental disorders which may be more susceptible to bioactive components in the diet through gene–environment interactions. While the current medical opinion is that soy products are healthy, accumulating evidence indicates adverse health effects in mouse and human models in response to conventionally grown, industrially processed, single-source, soy-based diets.

We hypothesize that consumption of high levels of soy protein isolate during postnatal development, through the use of single-source diets such as rodent chows and infant formula, is a dietary exposure that increases the risk of developing autism and obesity, particularly in vulnerable populations such as FXS. Mice for medical research are typically maintained in vivarium under controlled housing conditions with 24/7 access to a single-source diet. In an attempt to control experimental variables and reduce costs, those diets are typically grain-based rodent chows. There has been a movement in recent years to transition from soy/grain-based chows to low-phytoestrogen grain-based chows to avoid the confounding issue of bioactive soy phytoestrogens. For example, our standard vivarium chow was switched from Purina 5015 to Teklad 2019 in 2013. The effect of diet on experimental outcomes in rodent research is largely unexplored except for nutritional research where specific diets or supplements are under investigation. Diet–drug interactions are likely contributing to our current reproducibility crisis in biomedical research and have implications for both animal studies and human health. The human correlate of maintaining juvenile mice on a single, soy protein-based chow is exclusively feeding babies a soy protein-based infant formula. The evidence-based literature contains a dearth of studies investigating adverse health effects ensuing from consumption of soy-based infant formula, particularly in regard to developmental disabilities.

Prior rodent work in our laboratory indicates that soy/grain-based Purina 5015 chow increases susceptibility to audiogenic-induced seizures in mouse models of FXS (*Fmr1^KO^*), Alzheimer’s disease (Tg2576) and Down syndrome (Ts65Dn) [25] and exacerbates weight gain in both wild type (WT) and *Fmr1^KO^* mice [11,48] (Table 1). Retrospective medical record and survey analyses indicate that parental-reported use of soy-based infant formula is associated with increased incidence of seizures, autistic behaviors, allergies and gastrointestinal (GI) problems [23,49,50,51,52,53]. In addition, *Fmr1^KO^* mice exhibit increased weight gain when fed food pellets made from powdered soy-based infant formula compared to cow milk-based infant formula [11].

The objective of this study was to compare matched soy protein isolate- and casein protein-based purified ingredient diets, containing optimized macro- and micronutrient content for mice, on a panel of growth, behavioral and biomarker outcomes in *Fmr1^KO^* and littermate control mice. In a related paper, we assess brain protein biomarker expression by state-of-the-art mass spectrometry methodologies with the goal to define a proteomic signature induced by soy protein [54]. The long-term goal is to generate robust, reproducible data that identifies dietary biomarkers responsive to consumption of soy protein isolate and that forms a foundation for dietary recommendations to reduce the incidence of childhood autism and obesity.

## 2. Materials and Methods

### 2.1. Mouse Husbandry

Mice were obtained from our colony maintained at the University of Wisconsin–Madison for over 20 years; the *Fmr1^KO^* strain in the C57BL/6J background was originally provided by the laboratory of Dr. William Greenough (University of Illinois, Urbana-Champaign, IL, USA). *Fmr1^HET^* females and WT and *Fmr1^KO^* males used for breeding were transferred to test diets at least 12 days prior to mating. Breeding pairs were housed in microisolator cages on a 12 h (0600–1800) light cycle with ad libitum access to food and water (specific diets are described below). The bedding (Shepherd’s Cob + Plus, 1/4 inch cob) contained nesting material as the only source of environmental enrichment. Genotypes of offspring were determined by PCR analysis of DNA extracted from tail biopsies taken at weaning as previously described [15]. The animal study protocol was approved by the Institutional Animal Care and Use Committee (IACUC) at the University of Wisconsin, Madison (protocol code M005224).

### 2.2. Study Design

**Figure 1:**
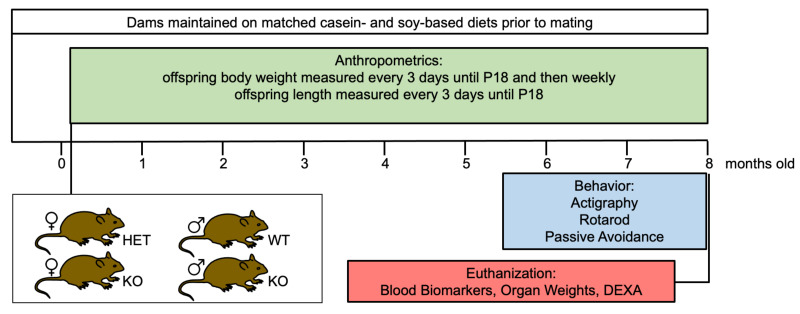
Study timeline. *Fmr1^HET^* and *Fmr1^KO^* female and WT and *Fmr1^KO^ male* mice were maintained on matched casein- and soy-based diets from conception and tested for growth, activity levels, motor coordination, learning and memory, blood-based amino acid and phytoestrogen levels, organ size, and bone density.

Study Design: The study design was a 2 × 2 factorial design with 2 main effects and interactions: diet (casein, soy) and genotype (WT or *Fmr1^HET^*, *Fmr1^KO^*). Sex was analyzed separately as there were large differences in body weight and activity between males and females. The study adhered to ARRIVE guidelines. The groups compared were *Fmr1^KO^* and littermate mice maintained on matched casein- and soy protein isolate-based purified ingredient diets containing AIN-93G-recommended levels of macro- and micronutrients. The experimental unit is a single animal. Mice from multiple litters were included in each cohort. The number of mice per cohort are stated in the figure legends and Table 2. Animals were excluded from an analysis if they died prematurely. Female *Fmr1^HET^* mice were randomly assigned to test diets for breeding purposes. The P.I. and staff were aware of diet group allocation throughout the study as diets were color coded pink (casein) and green (soy) to avoid mix-up of treatments over the 8-month dosing period. The P.I. and staff were blind to genotype identity during the postnatal anthropometric analysis of weight and length, but not later during behavioral assessments or analyses. Outcome measures assessed included the following: body weight and length during postnatal development until weaning; weekly body weight throughout the study; 24-7 activity levels in adults; motor coordination; learning and memory; blood-based amino acid, phytoestrogen and glucose levels; and organ measurements. The primary outcome measure was body weight. The statistical methods and software employed are included in the Methods below. A study timeline is provided in Figure 1.

### 2.3. Diets

The test diets were purified ingredient diets formulated by Envigo (Table S1). Casein-based TD.180374 is a modification of AIN-93G (Envigo TD.94045) with increased sodium at 2 g/kg diet (0.2%) to match the sodium content of soy protein isolate-based TD.180375. TD.180374 contains 17.7% protein by percent weight, 59.8% carbohydrate and 7.2% fat corresponding to 18.9%, 63.8% and 17.3%, respectively, % kcal with a total energy density of 3.7 kcal/g. Red food dye was added for visual differentiation. TD.180375 is modified from AIN-93G to replace casein with soy protein isolate and to match micronutrients to the control diet TD.180374 including 0.5% calcium, 0.3% available phosphorus, 0.2% sodium, 0.36% potassium, 0.3% chloride and 0.05% magnesium. TD.180375 contains 17.8% protein by % weight, 60.7% carbohydrate and 7.2% fat corresponding to 18.8%, 64.1% and 17.1%, respectively, % kcal with a total energy density of 3.8 kcal/g. Green food dye was added for visual differentiation. TD.180374 and TD.180375 were portioned into sealed Tupperware containers and stored at 4 °C prior to use. The vivarium standard chow was Teklad 2019 (Envigo, Fitchburg, WI, USA), which is a fixed formula diet with a nutritional profile of 19.0% protein, 9.0% fat, 2.6% fiber, 12.1% neutral detergent fiber, 5.0% ash, and 44.9% carbohydrate. The main ingredients are ground wheat, ground corn, corn gluten meal and wheat middlings. The energy density is 3.3 kcal/kg.

### 2.4. Growth Anthropometrics

Neonate mice were individually identified by tail tattoos at postnatal day 3 (P3) as previously described [55]. Pups were weighed every 3 days until weaning at P18 and thereafter once per week on an Ohaus Scout^TM^ Pro balance (OHAUS Corporation, Parsippany, NJ, USA). The length of the body was measured by gently flattening the pup on a sheet of graph paper on top of a hard surface and marking the tip of the nose and the bottom of the genital area; the distance in centimeters was later measured with a ruler by a blinded scientist and confirmed by a second scientist. Early mortalities were recorded. Pups were weaned at P18 with the ears notched for individual identification and tail biopsy samples collected for genotyping. Weaned mice were housed up to 4 per cage per IACUC rules. Data were analyzed with an ANOVA mixed-effects model and Tukey’s multiple comparison test using GraphPad Prism for macOS version 10.0.1 (170).

### 2.5. Actigraphy

Rest-activity rhythms were assessed as previously described [15,48,56]. Briefly, individually housed mice were tested under standard lighting conditions in home-made Plexiglas chambers containing passive infrared sensors mounted on the underside of the lids. The mice had ad libitum access to food and water. Each gross movement of the animal was recorded as an activity count with VitalView acquisition software version 4.1 (Mini Mitter Company, Inc., Bend, OR, USA). Activity counts were binned in 60 s epochs and scored on an activity scale (0–50) over a 9-day period. Data were retrieved with ACTIVIEW Biological Rhythm Analysis software version 1.3 (Mini Mitter Company, Inc.). One-minute activity epochs were averaged for the 7 full days of the recording period (excluding the first and last partial days). To calculate total average daily activity levels, data were summed over the 24 h period using Excel^®^ version 16.78.3 for Mac (v16.66.1). A chi-square periodogram method in ACTIVIEW was used to determine the diurnal rest-activity period over the 7 full days. Habituation was assessed by quantitating activity counts (binned into 15 min increments) during the first two hours after transitioning to the actigraphy caging. To assess activity as a function of the light/dark cycle, one-minute activity epochs were averaged for the full recording days and binned into 1 h and 4 h timeframes. Average data were plotted ± the standard error of the mean (SEM) and statistical significance determined by mixed-effects and 2-way ANOVA with Tukey’s multiple comparison test using GraphPad Prism for macOS version 10.0.1 (170).

### 2.6. Rotarod

Mice were acclimated to the test room for at least 20 min prior to testing on a Rotarod Treadmill (Med Associates Inc., Fairfax, VT, USA) as previously described [56]. The rotarod was set to a speed setting of 9, which accelerates from 4.0 to 40 rpm over 5 min. Mice were placed on the rotarod and the latency time of when the mouse fell off was recorded. If a mouse made two complete turns hanging onto the grip bar without actively walking/running, the mouse was counted as falling off of the beam. If more than 300 s elapsed, the mouse was removed from the beam. Experiments entailed four trials on day 1 and two trials on day 2. Data were analyzed by a 2-way ANOVA and Tukey’s multiple comparison test using GraphPad Prism version 10.0.1 (170).

### 2.7. Passive Avoidance

Passive Avoidance: Learning and memory were assessed as previously described [11,48,56]. Specifically, mice were acclimated to the experimental room for at least 20 min prior to testing in a foot shock passive avoidance paradigm using an aversive stimulator/scrambler (Med Associates Inc., St. Albans, VT, USA). A bench-top lamp was turned on behind the center of a light/dark shuttle box and aimed toward the back-left corner away from the dark side of the shuttle box. On the training day, a mouse was placed in the light side of the shuttle box toward the back corner away from the opening to the dark side of the shuttle box. The trap door in the shuttle box was open. After the mouse crossed over to the dark side, the trap door was closed and the latency time for the mouse to move from the light to the dark side was recorded. The mouse was allowed to equilibrate in the dark side for 5 s before receiving a foot shock (2 s, 0.6 mA). After 15 s, the mouse was removed from the shuttle box and returned to its home cage. The apparatus was cleaned with 70% EtOH between animals. At test times (6, 24 and 48 h after training), the mouse was placed in the light side of the shuttle box facing the left rear corner away from the opening to the dark side with the trap door open. After the mouse crossed to the dark side, the trap door was closed and the latency time for the mouse to move from the light to the dark side was recorded. If the mouse did not move to the dark side within 300 s, it was gently guided to the dark side and the trap door was closed. The mouse was allowed to equilibrate to the dark side for 5 s before return to the home cage. Mice received one shock on the training day; testing at 24 and 48 h measured extinction. Average data were plotted ±SEM and statistical significance determined by a 2-way ANOVA and Tukey’s multiple comparison test using GraphPad Prism for macOS version 10.0.1 (170).

### 2.8. Tissue Collection

Mice were weighed. Blood samples were collected from the abdominal artery under isoflurane anesthesia. Exsanguination euthanized the mouse. Blood glucose levels were assessed using a Precision Xtra blood glucose monitoring system (Abbott Diabetes Care Inc., Alameda, CA, USA). Low (LO) glucose meter readings were adjusted to 20 mg/dL. High (HI) off-scale glucose meter readings were adjusted to 500 mg/dL. Liver, abdominal adipose tissue and testes were dissected and weighed on an analytical balance. All samples were collected during the light phase after at least 4 h fasting. Individual datum points are plotted, with bars representing the mean of cohorts. Statistical significance was determined by a 2-way ANOVA with Tukey’s multiple comparison test using GraphPad Prism for macOS version 10.0.1 (170). Testes data were also analyzed by a 2-way ANOVA with Šídák’s multiple comparison test.

### 2.9. Amino Acid Analysis

Amino acids in blood plasma were quantified by ion-exchange chromatography on a Hitachi High-Technologies L-8900 Amino Acid Analyzer with a column (PF High SPE, 6.0 × 40 mm, Hitachi 855-4515, Tokyo, Japan) as previously described [57] with minor modifications [48]. Specifically, frozen plasma samples (50 μL each) were thawed and the samples mixed with 5 μL of 35% sulfosalicyclic acid (SSA) solution to achieve a 1:10 SSA to specimen ratio. Mixtures were vortexed for at least 10 s and spun at 14,000× *g* in a microcentrifuge for 3 min. Equal volumes (typically 30 μL each) of eluant and internal standard solution (4 nmol aminoethylcysteine diluted in water) were vortexed, and at least 50 μL of this mixture was transferred into a Hitachi sample vial (Hitachi ANO-2312) labeled with the specimen identification number and containing a 300 μL glass insert with poly-spring (Hitachi ANO-2313). Care was taken to avoid bubbles. In the case of bubbles, the insert was removed and briefly spun in a centrifuge to remove bubbles. The Hitachi vials were capped and loaded onto the ion exchange column (IEC) of the Hitachi Amino Acid analyzer. Amino acids were selectively eluted from the IEC with buffers of increasing pH with a programmed method of varied flow rates and temperatures. After elution, ninhydrin was mixed with the buffer–amino acid solution, heated to develop the purple color and read at 570 nm for the amino acids, phenylalanine and tyrosine. The total run time was 2.5 h per sample. The concentration of each amino acid was calculated by comparing the peak areas of the amino acid to the peak area of the internal standard, aminoethylcysteine, using Agilent OpenLAB software version A.04.07 Build 04.07.72. Degradation or major alterations of amino acids can occur with long-term storage. For example, plasma stored 7 months at −20 °C has slightly elevated aspartic acid, a large increase in glutamic acid, and no cystine compared with samples deproteinized and analyzed immediately; otherwise, all amino acids in fresh and stored frozen plasma agree within experimental error [58]. All samples were analyzed within 5 months of the date of collection. Statistical significance was determined by a 2-way ANOVA with Tukey’s multiple comparison test using GraphPad Prism for macOS version 10.0.1 (170).

### 2.10. Phytoestrogen Levels

Phytoestrogen levels in the feed were quantitated by NPAL N∙P Analytical Laboratories (St. Louis, MO, USA) using method reference code IFSP for the isoflavone profile, saponification assay. Results were reported in ppm for daidzin, daidzein, total daidzein compounds, genistin, genistein, total genistein compounds, glycitin, glycitein, total glycitein compounds, total isoflavones, daidzin (aglycone units), daidzein (aglycone units), total daidzein (aglycone units), genistin (aglycone units), genistein (aglycone units), total genistein (aglycone units), glycitin (aglycone units), glycitein (aglycone units), total glycitein (aglycone units), and total isoflavone (aglycone units). Statistical significance was determined by a 1-way ANOVA with Tukey’s multiple comparison test using GraphPad Prism for macOS version 10.0.1 (170).

Phytoestrogen levels in blood plasma were quantitated by liquid chromatography coupled to tandem mass spectrometry (LC/MS/MS) [59]. The genistein, biochanin A, S-equol, glycitein, and daidzein standards were purchased from Sigma (St. Louis, MO, USA). The daidzein-7-O-β-D-glucuronide, genistein-4-O-β-D-glucuronide, and R,S-equol-7-O-β-D-glucuronide standards were purchased from Toronto Research Chemicals (Toronto, ON, USA). The biochanin-A-7-O-β-D-glucuronide standard was purchased from Synthose (Concord, ON, USA). The internal standard, chrysin, was purchased from Sigma (St. Louis, MO, USA). All standards were dissolved in dimethyl sulfoxide (DMSO) at between 1 and 5 mg/mL before dilution to make the calibration curves. Calibration curves were prepared in potassium EDTA (KEDTA) mouse plasma (Innovative Research, Novi, MI, USA) that had been depleted of isoflavones by charcoal/dextran stripping. Standards were diluted into stripped plasma at between 0.25 and 100 ng/mL. Quality control samples for analytes were also prepared at between 3 and 30 ng/mL to verify instrument performance. All calibrators, quality control (QC) samples and unknown samples were prepared by precipitation and solid-phase extraction (SPE) processing on a Waters Ostro 96-well plate according to the manufacturer’s directions. The chrysin internal standard was spiked into the precipitation solution. After, SPE samples were dried and resuspended in 100 µL 20% acetonitrile/80% water/1 mM ammonium formate pH 5.0. For LC/MS/MS analysis, 5 µL of resuspended sample was injected onto a Waters HSS T3 2.1 × 150 mm column using a Waters I-Class Acquity UPLC system. Analytes were separated using a gradient of water/1 mM ammonium formate pH 5.0 (A solvent) and 98% acetonitrile/2% water/1 mM ammonium formate pH 5.0 (solvent B) flowing at 0.4 mL/min and the column temperature was held at 28 °C. All solvents were Optima LCMS grade from ThermoFisher Scientifics (Waltham, MA, USA). The gradient started at 12% B and changed linearly to 28% B at 0.85 min then to 75% B at 3.0 min then ramped to 95% B at 3.2 min. Solvent composition was held at 95% B until 4.1 min then ramped back to 12% B at 4.25 min. The total run-time for the LC gradient was 4.5 min. Analytes eluting from the column were introduced into a Sciex QTrap 5500 (AB Sciex LLC, Framingham, MA, USA) in negative ion mode and analyzed with a method divided into 6 periods. Spectrometer conditions for the source, fragmentation and detection of target compounds were optimized for compounds eluting during each period (see Table S2). Triplicate injections of sample calibrators and QC samples were analyzed for statistical purposes. Multiquant software version 3.03 (AB Sciex LLC, Framingham, MA, USA), was used to construct calibration curves fit to a quadratic model with 1/x^2^ weighting. Calibrators differing by more than 15% from the theoretical were eliminated from the model. For all assays, three of four QC samples were within 15% of their theoretical concentrations. Statistical significance was determined by a 1-way ANOVA with Tukey’s multiple comparison test using GraphPad Prism for macOS version 10.0.1 (170).

### 2.11. Bone Density

Mice were weighed and euthanized with CO_2_, the length of the mouse was measured from the tip of nose to base of the tail, and the mouse was scanned on a GE Lunar Piximus dual energy X-ray absorptiometry (DEXA) unit with an image area of 80 mm × 65 mm. The entire body minus the head, as the cranium has a very high bone density that could interfere with analysis of other bones, was imaged. Quality control scans using a phantom calibrator mouse were performed daily. Measurements included the following: bone mineral density (BMD, g/cm^2^), bone mineral content (BMC, g), bone area (cm^2^), total area (cm^2^), ratio soft tissue (RST), percent fat (%) and total tissue mass (TTM, g) for the whole body minus the head as analyzed with PIXI_MUS_ Lunar software version 1.43. Fat mass (g) was calculated by multiplying the percent fat by TTM. Lean mass (g) was calculated by subtracting fat mass from TTM mass. Statistical significance was determined by a 2-way ANOVA with Tukey’s multiple comparison test using GraphPad Prism for macOS version 10.0.1 (170).

## 3. Results

### 3.1. Postnatal Mortality

The number of live and dead pups were tracked over a 17-month period. The average number of live pups per litter were 6.95 ± 0.54 (SEM) (casein diet, 19 litters) and 6.28 ± 0.34 (soy diet, 29 litters) with no statistically significant difference (TTest, *p* = 0.27). For comparison, mice fed Teklad 2019 or Purina 5015 averaged 8 pups per litter. Pup deaths during the postnatal period prior to weaning were recorded. Pups found dead at or prior to P4 (predominantly at P2) included 32% (*n* = 14 dead, 30 live) WT/casein diet; 22% (*n* = 14 dead, 50 live) WT/soy diet, Chi statistic 0.25; 35% (*n* = 14 dead, 26 live) *Fmr1^HET^*/casein diet; 24% (*n* = 13 dead, 41 live) *Fmr1^HET^*/soy diet, Chi statistic 0.25; 19% (*n* = 18 dead, 76 live) *Fmr1^KO^*/casein diet; and 26% (*n* = 32 dead, 90 live) *Fmr1^KO^*/soy diet, Chi statistic 0.22. Thus, there were no statistically significant differences in death rates at P4 as a function of soy diet with a minimum of 40 mice per cohort. There were also no statistically significant differences based on genotype (casein: Chi statistic 0.092; soy: Chi statistic 0.8). Sex was not determined for pups that died at or before P4; however, due to the nature of the genetic mutation and the breeding strategy all, WT mice were males, all *Fmr1^HET^* mice were females, and *Fmr1^KO^* mice were either male or female. Note, three mice maintained on the soy diet died at or after P17 (one of each female *Fmr1^HET^*, female *Fmr1^KO^* and male *Fmr1^KO^*).

### 3.2. Phytoestrogen Levels in Diets

The levels of daidzin, daidzein, genistin, genistein, glycitin, glycitein, and their aglycone derivatives were determined in *n* = 16 (casein) and *n* = 20 (soy) batches of feed synthesized over a 38-month period and compared with *n* = 3 batches of Teklad 2019 and *n* = 4 batches of Purina 5015 (Figure S1). Daidzin, genistin, glycitin, total daizein, total genistein, total glycitein and total isoflavone compounds were significantly elevated in Purina 5015 chow > soy protein isolate-based purified ingredient diet > Teklad 2019 and casein-based purified ingredient diet. Daidzein, genistein and glycitein were significantly elevated in the soy protein isolate-based purified ingredient diet versus casein and Teklad 2019 and in Purina 5015 versus the casein diet. In addition, daidzein and genistein were higher in soy compared to Purina 5015. Aglycone derivatives followed the same trends.

### 3.3. Growth Metrics

**Figure 2:**
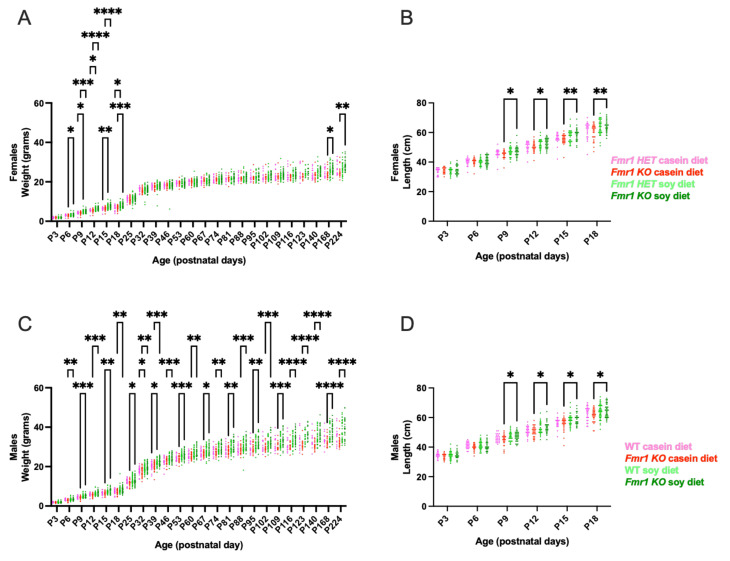
Growth metrics in response to soy protein isolate. Neonate mice were individually identified by tail tattoos at age P3 and measured every 3 days for body weight and body length until weaning at P18 and thereafter once per week for body weight. Data were analyzed with an ANOVA mixed-effects model and Tukey’s multiple comparison test. There were no outliers. (**A**) Body weight in *Fmr1^HET^* females fed casein diet (pink, *n* = 27), *Fmr1^KO^* females fed casein diet (red, *n* = 36), *Fmr1^HET^* females fed soy diet (light green, *n* = 40), and *Fmr1^KO^* females fed soy diet (dark green, *n* = 43): Time F (3.917, 379.6) = 4007, *p* < 0.0001; Diet/Genotype F (3, 142) = 2.398, *p* = 0.0706; Time × Diet/Genotype F (69, 2229) = 2.292, *p* < 0.0001; and Geisser–Greenhouse’s epsilon = 0.1703. (**B**) Body length in *Fmr1^HET^* females fed casein diet (pink, *n* = 23), *Fmr1^KO^* females fed casein diet (red, *n* = 31), *Fmr1^HET^* females fed soy diet (light green, *n* = 37), and *Fmr1^KO^* females fed soy diet (dark green, *n* = 39): Time F (3.208, 357.4) = 2644, *p* < 0.0001; Diet/Genotype F (3, 126) = 3.839, *p* = 0.0114; Time × Diet/Genotype F (15, 557) = 3.230, *p* < 0.0001; and Geisser–Greenhouse’s epsilon = 0.6417. (**C**) Body weight in WT males fed casein diet (pink, *n* = 30), *Fmr1^KO^* males fed casein diet (red, *n* = 38), WT males fed soy diet (light green, *n* = 49), and *Fmr1^KO^* males fed soy diet (dark green, *n* = 48): Time F (2.895, 315.0) = 5333, *p* < 0.0001; Diet/Genotype F (3, 161) = 15.64, *p* < 0.0001; Time × Diet/Genotype F (69, 2502) = 6.076, *p* < 0.0001; and Geisser–Greenhouse’s epsilon = 0.1259. (**D**) Body length in WT males fed casein diet (pink, *n* = 28), *Fmr1^KO^* males fed casein diet (red, *n* = 33), WT males fed soy diet (light green, *n* = 48), and *Fmr1^KO^* males fed soy diet (dark green, *n* = 45): Time F (4.021, 467.3) = 2932, *p* < 0.0001; Diet/Genotype F (3, 150) = 4.938, *p* = 0.0027; Time × Diet/Genotype F (15, 581) = 2.295, *p* = 0.0036; and Geisser–Greenhouse’s epsilon = 0.8042. * *p* < 0.05, ** *p* < 0.01, *** *p* < 0.001, **** *p* < 0.0001.

Body weight and length were measured every 3 days commencing at P3 until weaning at P18 and mice were weighed weekly thereafter until study completion at 8 months of age. There was a significant increase in body weight in *Fmr1^KO^* male mice maintained on the soy diet compared to casein at all time points commencing at P6 (Figure 2). WT mice exhibited increased body weight on soy P32–P39. There was a significant increase in body weight in *Fmr1^KO^* female mice maintained on soy diet compared to casein at P6–P18 and P168–P224 and in *Fmr1^HET^* female mice from P9–P18. Body length was measured from P3–P18 and increased in male and female *Fmr1^KO^* mice from P9–P18 with soy but not in WT male or *Fmr1^HET^* female mice. We find no statistically significant differences in body weight or length when comparing WT versus *Fmr1^KO^* males, or *Fmr1^HET^* versus *Fmr1^KO^* females, maintained on either diet. The maximal increase in body weight in response to the soy protein isolate was 16% in *Fmr1^HET^* female mice at P15, 21% in female *Fmr1^KO^* mice at P15, 11% in WT male mice at P18, and 20% in *Fmr1^KO^* male mice at P123 (Figure S2). Body weights in juvenile mice were on par with Jackson Laboratory data for C57BL/6J mice at 3 weeks of age (10.1 ± 1.7 SD females, 10.6 ± 1.9 SD males), which was the youngest age available. In adult animals, female mouse cohorts were on par with Jackson Laboratory data at 18 weeks of age (24.5 ± 2.6 SD); however, *Fmr1^KO^* males fed soy exceeded one standard deviation from the Jackson mean (33.3 ± 2.8 SD).

### 3.4. Activity Levels

**Figure 3:**
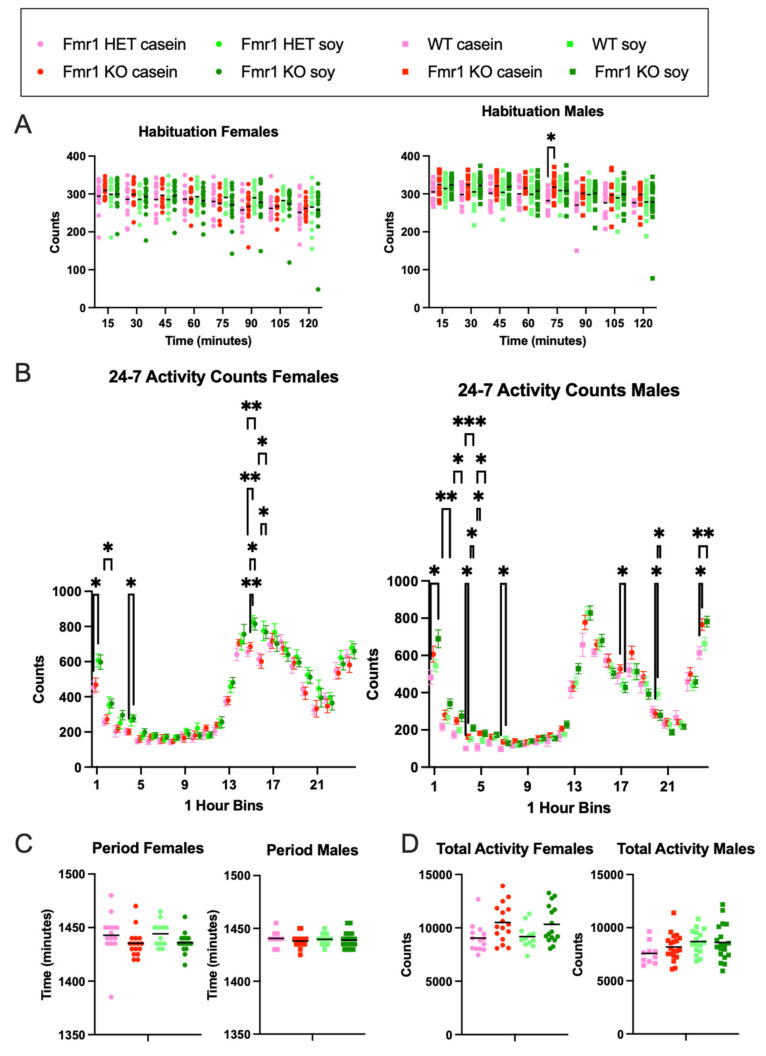
Actigraphy in response to *Fmr1* genotype and soy protein isolate. Rest-activity rhythms were assessed in adult mice. Each gross movement of the animal was recorded as an activity count for *Fmr1^HET^* females fed casein diet (pink, *n* = 14), *Fmr1^KO^* females fed casein diet (red, *n* = 12), *Fmr1^HET^* females fed soy diet (light green, *n* = 16), *Fmr1^KO^* females fed soy diet (dark green, *n* = 16), WT males fed casein diet (pink, *n* = 10), *Fmr1^KO^* males fed casein diet (red, *n* = 16), WT males fed soy diet (light green, *n* = 20), and *Fmr1^KO^* males fed soy diet (dark green, *n* = 19). Data were analyzed with an ANOVA 2-way model and Tukey’s multiple comparison test. (**A**) Habituation activity counts during first 2 h binned into 15 min increments in females: Time × Genotype/Diet F (21, 378) = 1.405, *p* = 0.1111; Time F (4.583, 247.5) = 19.42, *p* < 0.0001; Genotype/Diet F (3, 54) = 0.3215, *p* = 0.8098; Subject F (54, 378) = 15.33, *p* < 0.0001; and Geisser–Greenhouse’s epsilon = 0.6547. Habituation activity counts during first 2 h binned into 15 min increments in males: Time × Genotype/Diet F (21, 427) = 0.9786, *p* = 0.4889; Time F (5.078, 309.8) = 18.48, *p* < 0.0001; Genotype/Diet F (3, 61) = 2.489, *p* = 0.0688; Subject F (61, 427) = 9.029, *p* < 0.0001; and Geisser–Greenhouse’s epsilon = 0.7254. (**B**) 24-7 activity counts binned in 1 h increments in females: Bin × Genotype/Diet F (69, 1242) = 1.348, *p* = 0.0331; Bin F (11.36, 613.2) = 221.5, *p* < 0.0001; Genotype/Diet F (3, 54) = 3.517, *p* = 0.0211; Subject F (54, 1242) = 8.292, *p* < 0.0001; and Geisser–Greenhouse’s epsilon = 0.4937. Counts of 24-7 activity binned in 1 h increments in males: Bin × Genotype/Diet F (69, 1403) = 2.676, *p* < 0.0001; Bin F (10.46, 637.9) = 290.3, *p* < 0.0001; Genotype/Diet F (3, 61) = 1.755, *p* = 0.1653; Subject F (61, 1403) = 8.401, *p* < 0.0001; and Geisser–Greenhouse’s epsilon = 0.4547. (**C**) Period in females: Interaction F (1, 54) = 0.008289, *p* = 0.9278; Genotype F (1, 54) = 0.06620, *p* = 0.7979; and Diet F (1, 54) = 4.401, *p* = 0.0406. Period in males: Interaction F (1, 61) = 0.2856, *p* = 0.5950; Genotype F (1, 61) = 0.001677, *p* = 0.9675; and Diet F (1, 61) = 0.9682, *p* = 0.3290. (**D**) Total activity in females: Interaction F (1, 54) = 0.1423, *p* = 0.7075; Genotype F (1, 54) = 0.001610, *p* = 0.9681; and Diet F (1, 54) = 10.28, *p* = 0.0023. Total activity in males: Interaction F (1, 61) = 0.9849, *p* = 0.3249; Genotype F (1, 61) = 4.881, *p* = 0.0309; Diet F (1, 61) = 0.5408, *p* = 0.4649. * *p* < 0.05, ** *p* < 0.01, *** *p* < 0.001.

Activity levels were measured 24-7 over a nine-day period. Habituation to the novel chambers was assessed during the first 2 h of the first recording day with no statistical differences in activity as a function of genotype or diet in females (Figure 3A). In males, there was increased activity in *Fmr1^KO^* mice compared to the WT fed casein diet at the beginning of hour 2 suggesting faster acclimation to the chambers in WT male mice on casein. Total 24-7 activity counts over 7 full recording days were not statistically different as a function of the genotype or diet in males or females (Figure 3D). However, binning the data hourly indicated statistical differences during the beginning of the light phase as well as during the acrophase, i.e., the time within the 24 h day when peak activity occurs (Figure 3B and Figure S3). *Fmr1^HET^* female mice were 34% more active during the first hour of the light cycle when fed soy; *Fmr1^KO^* females were 38% more active during the 4th hour of the light cycle when fed soy; *Fmr1^HET^* and *Fmr1^KO^* females were 26% and 19%, respectively, more active during hour 15 of the dark cycle when fed soy; and *Fmr1^KO^* females were 28% more active at hour 16 when fed soy. The acrophase for females was hour 18 for *Fmr1^HET^* fed casein, hour 17 for *Fmr1^KO^* fed casein, and hour 15 for *Fmr1^HET^* and *Fmr1^KO^* fed soy. Of note, there were twin activity peaks during the dark cycle with females fed casein with the slightly lower peak at hours 15 and 14, respectively, for *Fmr1^HET^* and *Fmr1^KO^*. *Fmr1^KO^* male mice exhibited 64% and 70% increased activity compared to WT at hours 4 and 5, respectively, of the light cycle in response to casein and 40% at hour 4 with the soy diet; WT mice exhibited 57% increased activity at hour 7 on soy compared to casein. During the dark cycle, *Fmr1^KO^* exhibited 19% decreased activity at hour 17 with soy versus casein, 29% decreased activity at hour 20 compared to WT on soy, and 25% increased activity at hour 24 compared to WT on casein. The acrophase was at hour 14 for all male mice irrespective of genotype or diet. The period was not statistically different as a function of genotype or diet in males or females (Figure 3C). Overall, the data indicated increased activity in female *Fmr1^HET^* and *Fmr1^KO^* mice in response to soy during the first half of the light cycle and at acrophase of the dark cycle whereas genotype-specific effects were apparent in the males with increased activity in the *Fmr1^KO^* during the first half of the light cycle and decreased activity during the second half of the dark cycle.

### 3.5. Behavior

**Figure 4:**
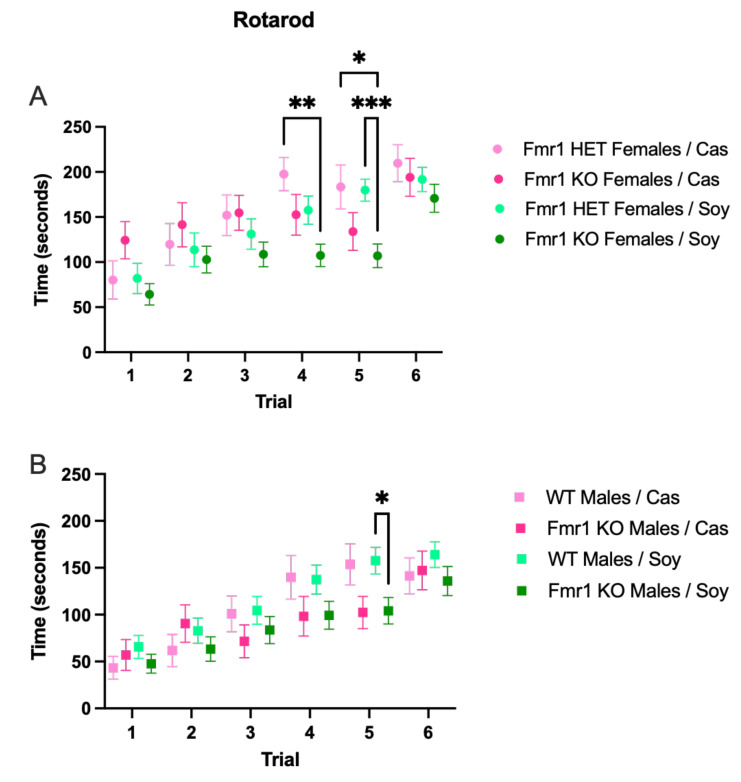
Motor coordination in response to *Fmr1* genotype and soy protein isolate. Adult mice were tested for motor coordination by measuring run latency times on a rotarod. Data were analyzed with an ANOVA 2-way model and Tukey’s multiple comparison test. (**A**) Run latency times in *Fmr1^HET^* females fed casein diet (pink, *n* = 16), *Fmr1^KO^* females fed casein diet (red, *n* = 17), *Fmr1^HET^* females fed soy diet (light green, *n* = 25), and *Fmr1^KO^* females fed soy diet (dark green, *n* = 28): Trial F (4.551, 373.2) = 24.07, *p* < 0.0001; Diet/Genotype F (3, 82) = 3.367, *p* = 0.0225; Trial × Diet/Genotype F (15, 410) = 1.678, *p* = 0.0525; Subject F (82, 410) = 4.347, *p* < 0.0001; and Geisser–Greenhouse’s epsilon = 0.9102. (**B**) Run latency times in WT males fed casein diet (pink, *n* = 14), *Fmr1^KO^* males fed casein diet (red, *n* = 20), WT males fed soy diet (light green, *n* = 32), and *Fmr1^KO^* males fed soy diet (dark green, *n* = 28): Trial F (4.322, 389.0) = 37.29, *p* < 0.0001; Diet/Genotype F (3, 90) = 1.390, *p* = 0.2511; Trial × Diet/Genotype F (15, 450) = 1.428, *p* = 0.1300; Subject F (90, 450) = 7.506, *p* < 0.0001; and Geisser–Greenhouse’s epsilon = 0.8644. * *p* < 0.05, ** *p* < 0.01, *** *p* < 0.001.

There were trends for decreased running time on the rotarod in *Fmr1^KO^* mice compared to littermate controls on the soy diet for all trials in both sexes (Figure 4), with the only statistically significant difference at Trial 5 with improved motor coordination in *Fmr1^HET^* versus *Fmr1^KO^* females (Figure 4A) and in WT versus *Fmr1^KO^* males on the soy diet (Figure 4B). There were no statistically significant differences in motor coordination assessed by the rotarod assay as a function of the *Fmr1* genotype, diet and trial by 3-way ANOVA for males or females (Figure S4). There are trends for increased motor coordination in females versus males, which is consistent with the decreased body weight in females. Analyses of individual trials by 1-way ANOVA indicated a sex-specific difference in *Fmr1^KO^* mice fed casein with improved motor coordination in females versus males during trial 3 (Figure S4).

**Figure 5:**
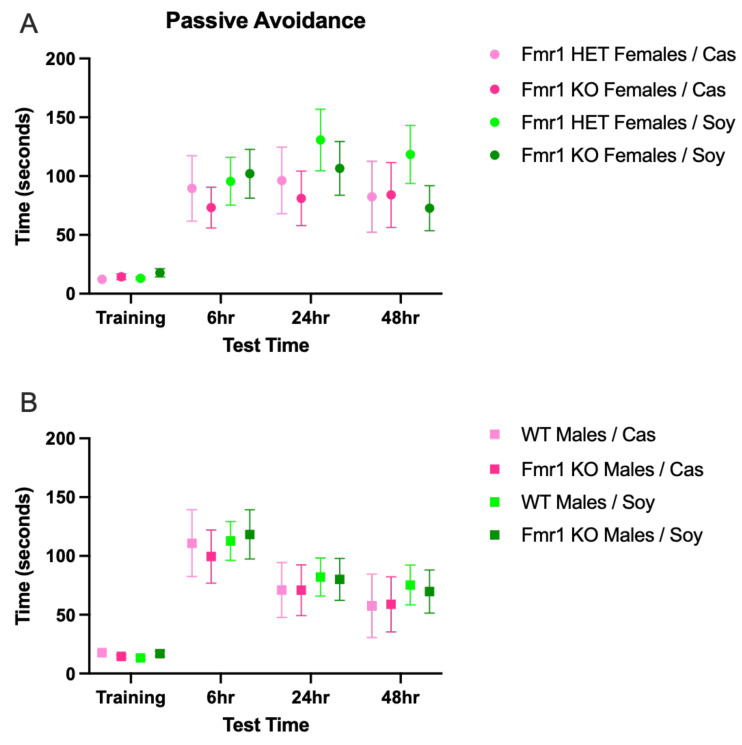
Learning and memory in response to *Fmr1* genotype and soy protein isolate. Adult mice were tested for learning and memory in the passive avoidance task. Data were analyzed with an ANOVA 2-way model and Tukey’s multiple comparison test. (**A**) Light to dark side latency times in *Fmr1^HET^* females fed casein diet (pink, *n* = 16), *Fmr1^KO^* females fed casein diet (red, *n* = 17), *Fmr1^HET^* females fed soy diet (light green, *n* = 25), and *Fmr1^KO^* females fed soy diet (dark green, *n* = 28): Trial F (2.524, 206.9) = 24.55, *p* < 0.0001; Diet/Genotype F (3, 82) = 0.4994, *p* = 0.6837; Trial × Diet/Genotype F (9, 246) = 0.7249, *p* = 0.6859; Subject F (82, 246) = 3.867, *p* < 0.0001; and Geisser–Greenhouse’s epsilon = 0.8412. (**B**) Light to dark side latency times in WT males fed casein diet (pink, *n* = 14), *Fmr1^KO^* males fed casein diet (red, *n* = 20), WT males fed soy diet (light green, *n* = 32), and *Fmr1^KO^* males fed soy diet (dark green, *n* = 28): Trial F (2.672, 240.5) = 29.49, *p* < 0.0001; Diet/Genotype F (3, 90) = 0.1501, *p* = 0.9294; Trial × Diet/Genotype F (9, 270) = 0.1211, *p* = 0.9992; Subject F (90, 270) = 3.534, *p* < 0.0001; and Geisser–Greenhouse’s epsilon = 0.8906.

There were no statistically significant differences in learning and memory as a function of genotype, sex or diet (Figure 5), albeit there were trends for increased learning in male mice and increased memory in female mice (Figure S5). There were also trends for increased memory at the 24 h test point irrespective of genotype in mice fed soy compared to the casein diet.

### 3.6. Blood Biomarkers

**Figure 6:**
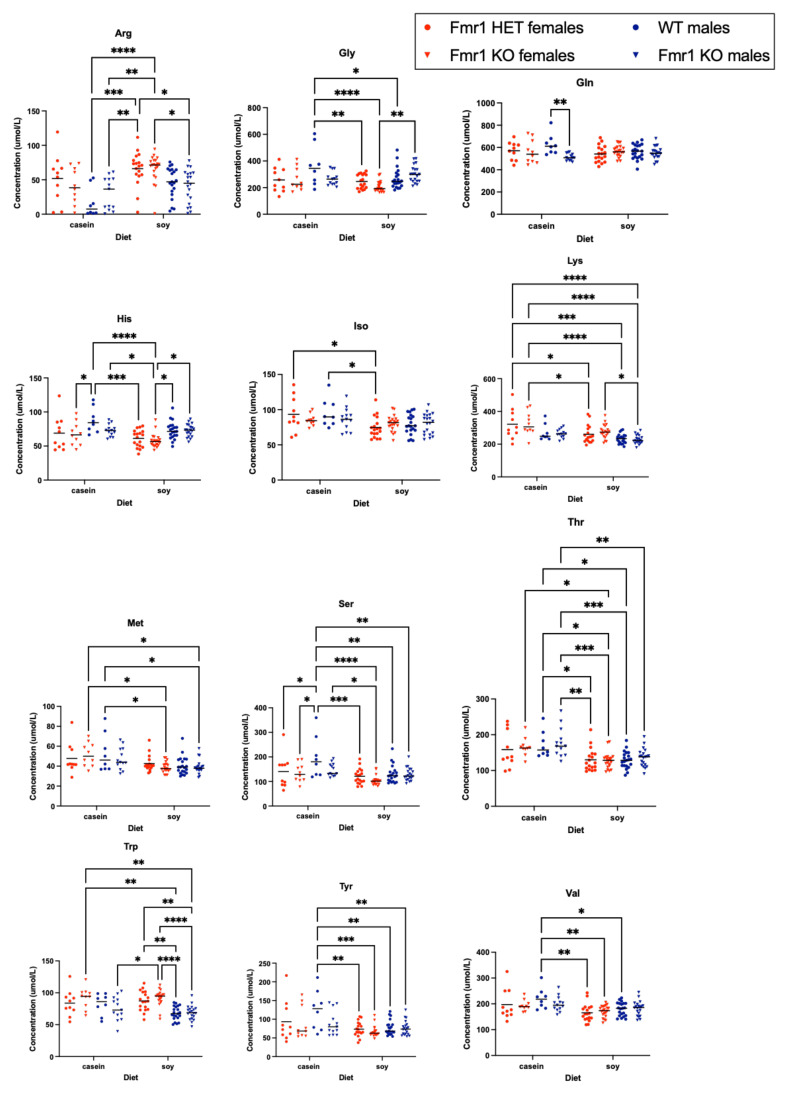
Blood-based amino acid levels in response to sex, *Fmr1* genotype and soy protein isolate. Blood amino acid levels were quantitated as a function of genotype and diet. Data were analyzed with an ANOVA 2-way model and Tukey’s multiple comparison test. Amino acids with significant changes are presented here. Remaining amino acids as well as BCAA and ratios of specific amino acids to BCAA are provided in Supplementary Figure S6. * *p* < 0.05, ** *p* < 0.01, *** *p* < 0.001, **** *p* < 0.0001.

The levels of several amino acids were altered in blood plasma as a function of genotype and/or diet (Figure 6 and Figure S6). Comparing casein versus soy diets: *Fmr1^HET^* females fed soy had decreased isoleucine and lysine levels; WT male mice exhibited significantly lower levels of glycine, serine, threonine, tyrosine and valine; *Fmr1^KO^* females had decreased methionine and threonine levels; and *Fmr1^KO^* males had lower threonine levels. Comparing genotypes, 3-methylhistidine was elevated in *Fmr1^KO^* males compared to females with both casein and soy diets. WT male mice on the casein diet showed elevated glutamine compared to *Fmr1^KO^* mice on the casein diet; the effect was lost with the soy diet. Selectively with the soy diet: tryptophan was elevated in *Frm1^HET^* and *Fmr1^KO^* females compared to WT and *Fmr1^KO^* males; glycine and histidine were reduced in *Fmr1^KO^* females compared to males; and arginine and lysine were increased in *Fmr1^KO^* females compared to males.

**Figure 7:**
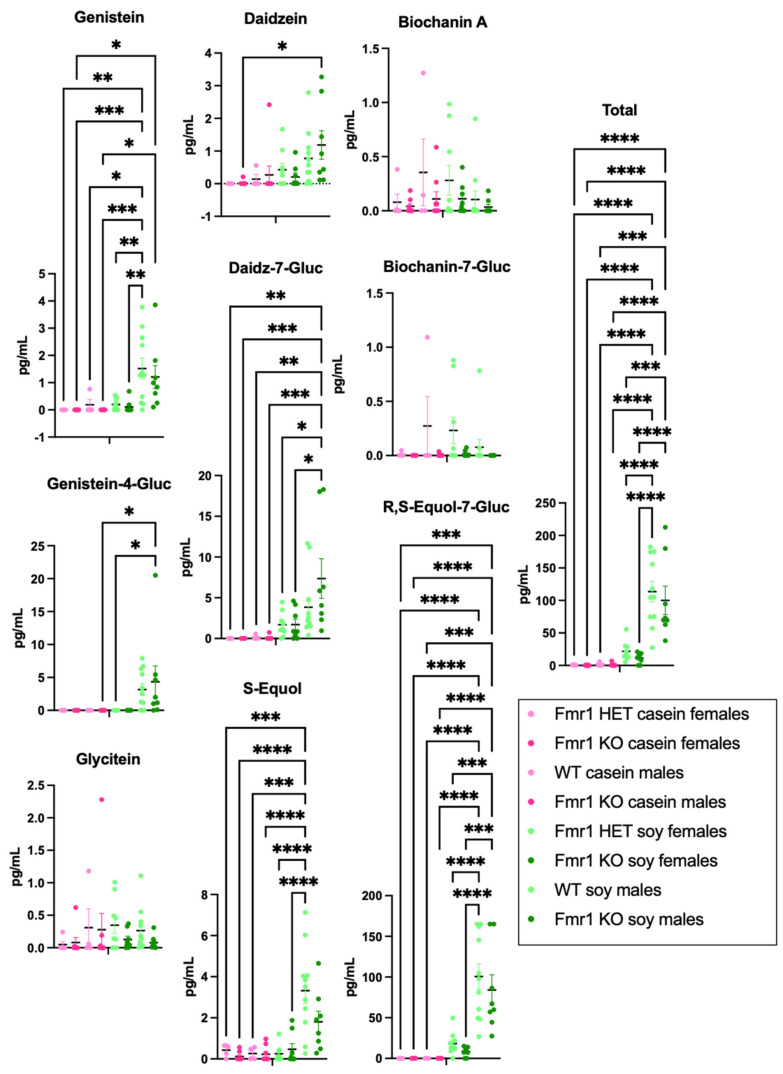
Blood-based phytoestrogen levels in response to sex, *Fmr1* genotype and soy protein isolate. Blood phytoestrogen levels were quantitated as a function of sex, genotype and diet in 4-month-old mice fed casein versus soy diets. Data were analyzed with an ANOVA 2-way model and Tukey’s multiple comparison test. Complementary data presented in Supplementary Figure S7 include blood samples from the same mice that underwent behavioral and amino acid analyses; however, due to lockdown of the testing laboratory during COVID restrictions, there were problems with sample degradation during prolonged freezer storage. * *p* < 0.05, ** *p* < 0.01, *** *p* < 0.001, **** *p* < 0.0001.

The levels of 9 phytoestrogens (genistein, genistein-4-O-β-D-glucuronide, glycitein, daidzein, daidzein-7-O-β-D-glucuronide, S-equol, R,S-equol-7-O-β-D-glucuronide, biochanin A, and biochanin-A-7-O-β-D-glucuronide) were quantitated in blood plasma from the same mice that underwent behavioral and amino acid analyses; however, due to lockdown of the testing laboratory during COVID restrictions, there were problems with sample degradation during prolonged freezer storage. Nonetheless, there were elevated levels of S-equol in plasma from WT male mice fed the soy diet compared to *Fmr1^HET^* and *Fmr1^KO^* female mice fed the soy diet, and compared to all mice fed the casein diet, (Figure S7). In addition, total phytoestrogen levels were significantly higher in WT male mice fed soy compared to *Fmr1^HET^* and *Fmr1^KO^* females fed soy. The most predominant plasma phytoestrogen was R,S-equol-7-O-β-D-glucuronide with averages of 25.01 and 27.45 ng/mL in WT and *Fmr1^KO^* males, respectively, fed the soy diet. New cohorts of animals were generated, euthanized at age 4 months, and blood plasma was collected for phytoestrogen analyses (Figure 7). Trends were the same as the degraded samples albeit average glycitein, S-equol and biochanin A levels were lower and genistein-4-O-β-D-glucuronide, daidzein-7-O-β-D-glucuronide and R,S-equol-7-O-β-D-glucuronide levels were higher with the new samples, which could be due to altered metabolism in the younger age mice. Genistein, genistein-4-O-β-D-glucuronide, daidzein-7-O-β-D-glucuronide and R,S-equol-7-O-β-D-glucuronide were significantly higher in male *Fmr1^KO^* mice on soy compared to the casein diet. Genistein, S-equol and R,S-equol-7-O-β-D-glucuronide were higher in WT male mice on soy versus casein. S-equol was higher in WT male versus *Fmr1^HET^* female mice on soy. R,S-equol-7-O-β-D-glucuronide was higher in WT and *Fmr1^KO^* male versus *Fmr1^HET^* and *Fmr1^KO^* female mice, respectively, on soy. Daidzein-7-O-β-D-glucuronide was higher in male versus female *Fmr1^KO^* mice fed soy. Total phytoestrogen levels were primarily attributed to R,S-equol-7-O-β-D-glucuronide, which was at least 15-fold higher than the other phytoestrogens.

### 3.7. Organ Measurements

**Figure 8:**
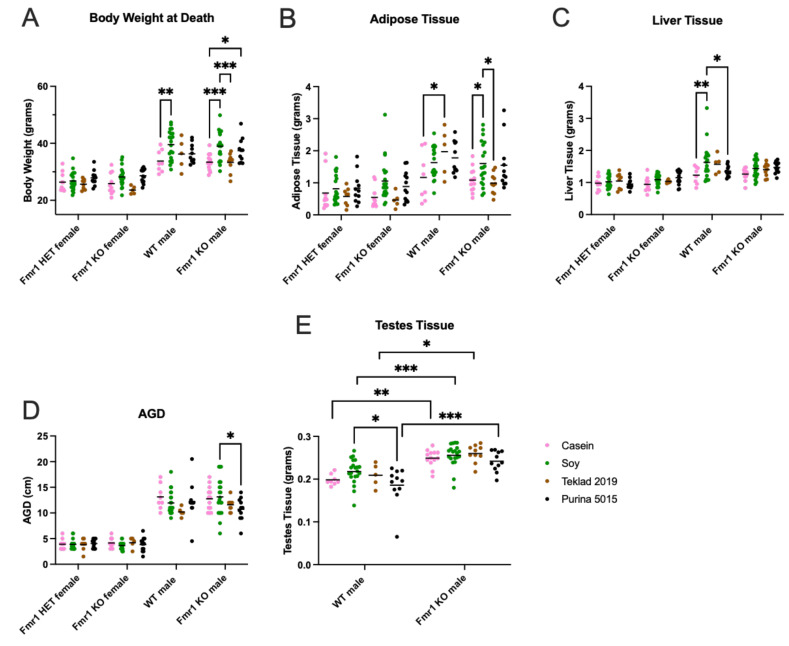
Organ measurements in response to sex, *Fmr1* genotype and soy protein isolate. Mice were weighed and euthanized after completion of the behavioral studies and organ weights measured for *Fmr1^HET^* females fed casein diet (pink, *n* = 10), soy diet (green, *n* = 19), Teklad 2019 (brown, *n* = 8), or Purina 5015 (black, *n* = 11); *Fmr1^KO^* females fed casein diet (pink, *n* = 10), soy diet (green, *n* = 22), Teklad 2019 (brown, *n* = 5) or Purina 5015 (black, *n* = 13); WT males fed casein diet (pink, *n* = 8), soy diet (green, *n* = 21), Teklad 2019 (brown, *n* = 5), or Purina 5015 (black, *n* = 11); and *Fmr1^KO^* males fed casein diet (pink, *n* = 13), soy diet (green, *n* = 20), Teklad 2019 (brown, *n* = 10), or Purina 5015 (black, *n* = 11). Data were analyzed with an ANOVA 2-way model and Tukey’s multiple comparison test. (**A**) Body weight at euthanization: Interaction F (9, 181) = 1.734, *p* = 0.0842; Genotype F (3, 181) = 89.54, *p* < 0.0001; and Diet F (3, 181) = 11.83, *p* < 0.0001. (**B**) Adipose tissue weight: Interaction F (9, 181) = 1.350, *p* = 0.2142; Genotype F (3, 181) = 26.57, *p* < 0.0001; and Diet F (3, 181) = 6.376, *p* = 0.0004. (**C**) Liver tissue weight: Interaction F (9, 181) = 1.328, *p* = 0.2252; Genotype F (3, 181) = 29.80, *p* < 0.0001; and Diet F (3, 181) = 4.755, *p* = 0.0032. (**D**) AGD: Interaction F (9, 181) = 1.538, *p* = 0.1377; Genotype F (3, 181) = 218.7, *p* < 0.0001; and Diet F (3, 181) = 1.852, *p* = 0.1393. (**E**) Testes weight for WT males: Interaction F (3, 91) = 0.6004, *p* = 0.6164; Genotype F (1, 91) = 64.29, *p* = 0.0001; and Diet F (3, 91) = 3.622, *p* = 0.0160. * *p* < 0.05, ** *p* < 0.01, *** *p* < 0.001.

Body weight was assessed immediately prior to euthanization and compared with male mice maintained on two standard mouse chows (Teklad 2019 and Purina 5015) (Figure 8). WT mice fed the soy diet weighed 17% more than WT mice fed the casein diet. *Fmr1^KO^* male mice fed soy weighed 17% more than *Fmr1^KO^* male mice fed the casein diet or Teklad 2019. *Fmr1^KO^* male mice fed Purina 5015 weighed 12% more than *Fmr1^KO^* mice fed the casein diet. WT male mice had 68% elevated adipose tissue when fed Teklad 2019 compared to the casein diet. *Fmr1^KO^* male mice fed soy had 48% elevated adipose tissue compared to casein and 62% compared to Teklad 2019. Liver weight was elevated 33% in WT male mice fed soy compared to casein and 20% in soy versus Purina 5015. *Fmr1^KO^* male mice had a 22% increase of length in angiogenital distance (AGD) when fed soy compared to Purina 5015. Testes weight was the single phenotype under study that was statistically significantly higher in *Fmr1^KO^* compared to WT mice irrespective of diet (18–30% increase). WT male mice fed soy had 49% elevated glucose compared to the casein diet (Figure S8). Of note, abdominal disease, primarily steatosis, was observed in 32% of mice fed the casein diet compared to 6.1% soy, 7.1% Teklad 2019 and 2.2% Purina 5015 (Tables S3–S5). There were no statistically significant differences in organ measurements in female mice as a function of genotype or diet.

**Figure 9:**
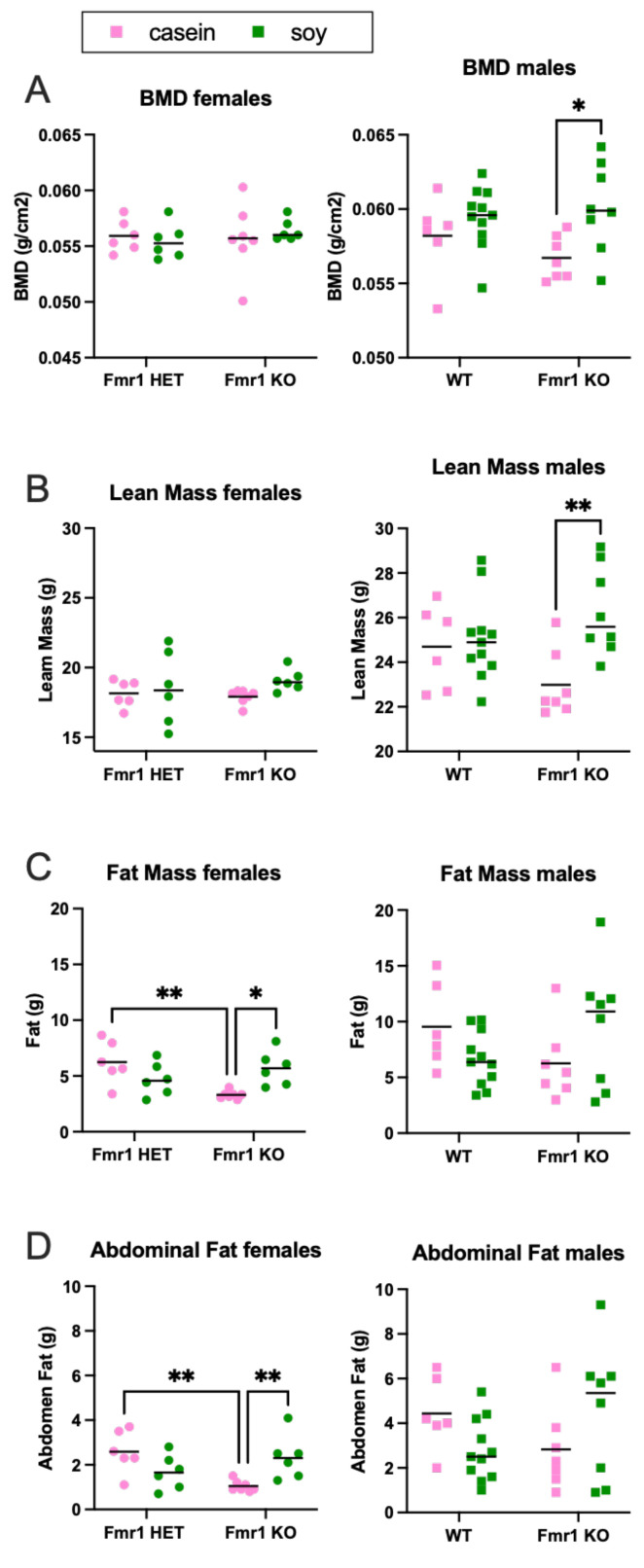
DEXA analysis in response to soy protein isolate in 8-month-old mice. Mice were scanned in a GE Lunar Piximus DEXA unit after euthanization at 8 months of age. *Fmr1^HET^* (*n* = 6 casein, *n* = 6 soy) and *Fmr1^KO^* (*n* = 7 casein, *n* = 6 soy) females (denoted by circles) and WT (*n* = 6 casein, *n* = 11 soy) and *Fmr1^KO^* (*n* = 7 casein, *n* = 8 soy) males (squares) were analyzed separately with an ANOVA mixed-effects model and Tukey’s multiple comparison test in response to casein (pink) and soy (green) diets. (**A**) BMD females: Interaction F (1, 21) = 0.5401, *p* = 0.4705; Genotype F (1, 21) = 0.2170, *p* = 0.6462; and Diet F (1, 21) = 0.02411, *p* = 0.8781. BMD males: Interaction F (1, 28) = 1.649, *p* = 0.2097; Genotype F (1, 28) = 0.2190, *p* = 0.6434; and Diet F (1, 28) = 7.577, *p* = 0.0103. (**B**) Lean mass females: Interaction F (1, 21) = 0.4748, *p* = 0.4983; Genotype F (1, 21) = 0.07518, *p* = 0.7868; and Diet F (1, 21) = 1.780, *p* = 0.1964. Lean mass males: Interaction F (1, 28) = 4.926, *p* = 0.0347; Genotype F (1, 28) = 0.1325, *p* = 0.7186; and Diet F (1, 28) = 7.609, *p* = 0.0101. (**C**) Fat mass females: Interaction F (1, 21) = 12.25, *p* = 0.0021; Genotype F (1, 21) = 3.012, *p* = 0.0973; and Diet F (1, 21) = 0.5993, *p* = 0.4475. Fat mass males: Interaction F (1, 28) = 5.021, *p* = 0.0332; Genotype F (1, 28) = 0.01977, *p* = 0.8892; and Diet F (1, 28) = 0.01960, *p* = 0.8897. (**D**) Abdominal fat females: Interaction F (1, 21) = 12.44, *p* = 0.0020; Genotype F (1, 21) = 1.950, *p* = 0.1772; and Diet F (1, 21) = 0.3568, *p* = 0.5567. Abdominal fat males: Interaction F (1, 28) = 5.102, *p* = 0.0319; Genotype F (1, 28) = 0.005382, *p* = 0.9420; and Diet F (1, 28) = 0.001187, *p* = 0.9728. Complementary data presented in Supplementary Figure S9 includes the following: BMC, bone area, % fat, TTM, RST, total area, body weight and body length. * *p* < 0.05, ** *p* < 0.01.

**Figure 10:**
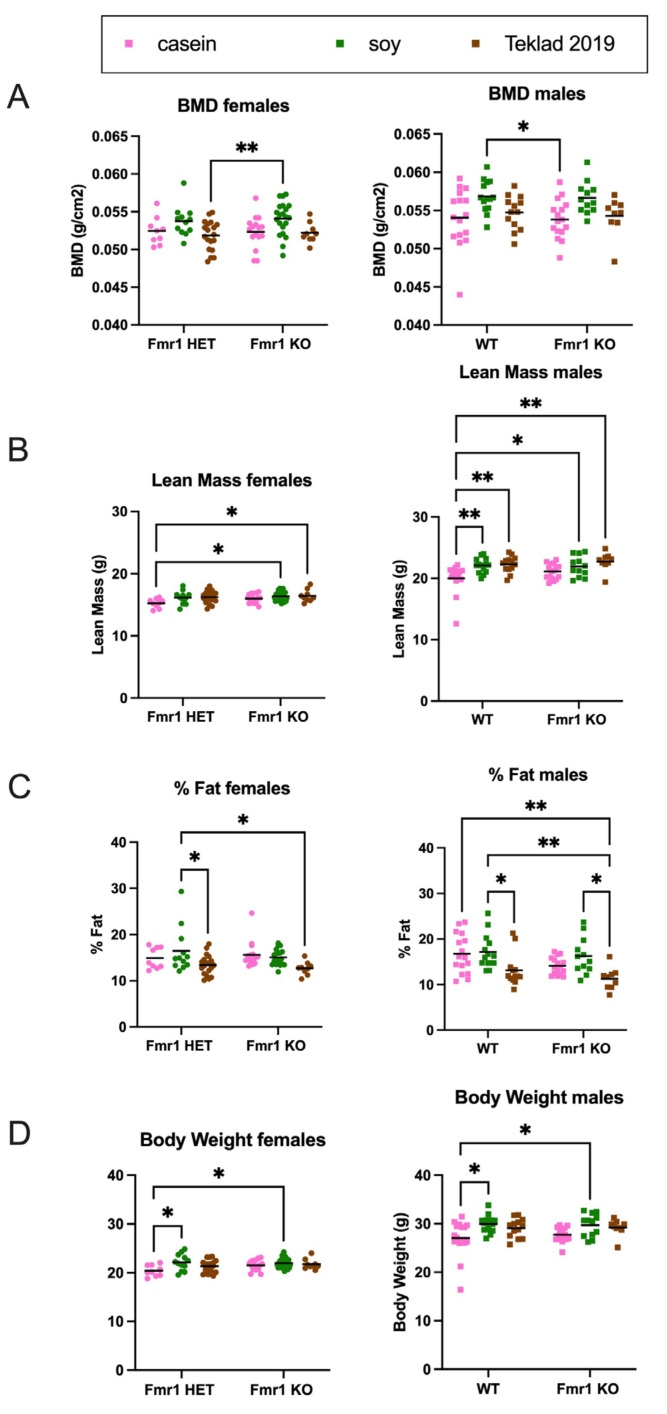
DEXA analysis in response to soy protein isolate in 4-month-old mice. Mice were scanned in a GE Lunar Piximus DEXA unit after euthanization at 4 months of age. *Fmr1^HET^* (*n* = 9 casein, *n* = 12 soy, *n* = 19 Teklad 2019) and *Fmr1^KO^* (*n* = 16 casein, *n* = 21 soy, *n* = 9 Teklad 2019) females (circles) and WT (*n* = 16 casein, *n* = 14 soy, *n* = 13 Teklad 2019) and *Fmr1^KO^* (*n* = 16 casein, *n* = 12 soy, *n* = 9 Teklad 2019) males (squares) were analyzed separately with an ANOVA mixed-effects model and Tukey’s multiple comparison test in response to casein (pink), soy (green) and Teklad 2019 (brown) diets. (**A**) BMD females: Interaction F (2, 80) = 0.1103, *p* = 0.8957; Genotype F (2, 80) = 7.038, *p* = 0.0015; and Diet F (1, 80) = 0.1810, *p* = 0.6716. BMD males: Interaction F (2, 74) = 0.01467, *p* = 0.9854; Genotype F (2, 74) = 8.327, *p* = 0.0005; and Diet F (1, 74) = 0.2385, *p* = 0.6267. (**B**) Lean mass females: Interaction F (2, 80) = 0.7909, *p* = 0.4569; Genotype F (2, 80) = 4.958, *p* = 0.0093; and Diet F (1, 80) = 3.841, *p* = 0.0535. Lean mass males: Interaction F (2, 74) = 1.052, *p* = 0.3545; Genotype F (2, 74) = 10.95, *p* < 0.0001; and Diet F (1, 74) = 1.631, *p* = 0.2055. (**C**) % Fat females: Interaction F (2, 80) = 1.025, *p* = 0.3633; Genotype F (2, 80) = 7.343 *p* = 0.0012; and Diet F (1, 80) = 0.5956, *p* = 0.4426. % Fat males: Interaction F (2, 74) = 0.4750, *p* = 0.6238; Genotype F (2, 74) = 10.28, *p* = 0.0001; and Diet F (1, 74) = 5.071, *p* = 0.0273. (**D**) Body weight females: Interaction F (2, 80) = 1.746, *p* = 0.1810; Genotype F (2, 80) = 5.422, *p* = 0.0062; and Diet F (1, 80) = 2.737, *p* = 0.1019. Body weight males: Interaction F (2, 74) = 0.2465, *p* = 0.7822; Genotype F (2, 74) = 8.373, *p* = 0.0005; and Diet F (1, 74) = 0.1204, *p* = 0.7296. Complementary data presented in Supplementary Figure S10 includes the following: BMC, bone area, TTM, RST, total area, body length and rectal temperature. * *p* < 0.05, ** *p* < 0.01.

The increased body weight in mice as a function of the soy diet could be due to increased fat mass, lean mass or a combination. DEXA analysis at 8 months of age indicated increased fat mass and total body area in *Fmr1^KO^* females and increased lean mass and BMD in *Fmr1^KO^* males on the soy diet (Figure 9 and Figure S9), suggesting soy-dependent, sex-specific differences. There was also a genotype-specific difference in female mice maintained on the casein diet, where *Fmr1^HET^* had significantly higher fat mass than *Fmr1^KO^* females. Of interest, the DEXA fat mass and TTM data did not concur with abdominal adipose tissue and whole-body weights, respectively (Figure 8), in *Fmr1^KO^* male mice in response to soy nor did DEXA fat mass agree with abdominal adipose tissue weight in females. We questioned if differential distribution of adipose tissue across the body was contributing to the varied findings. Hence, we drew new regions of interest (ROIs), including only the abdomen, and quantitated abdominal fat with the PIXI_MUS_ Lunar software version 1.43 (Figure 9D). The results mirrored the whole-body DEXA data. For the *Fmr1^KO^* males fed casein versus soy, the TTM ANOVA analysis just missed statistical significance at *p* = 0.0581, but overall, it remains to be determined why fat masses assessed by the two methods do not agree. DEXA analysis at 4 months of age indicated increased lean mass and TTM in male WT mice fed soy (Figure 10 and Figure S10). There was a genotype-specific difference in females with increased TTM in *Fmr1^HET^* mice fed soy compared to casein.

## 4. Discussion

### 4.1. Study Findings in Context of the Scientific Literature

Rodent colonies for medical research are typically maintained on grain-based chows. Soybean meal is a major ingredient in classical chows such as Purina 5015, and the high phytoestrogen content has known effects on reproductive function, BMD, anxiety, and pain [60,61]. Here, we tested the effect of soy protein isolate in the context of a purified ingredient diet in a mouse model of FXS. Our experimental study was a side-by-side comparison of growth, activity, behavior, blood biomarker, and organ biomarkers in *Fmr1^KO^* mice as a function of matched casein and soy protein isolate diets where cohorts included both sexes and littermate controls. The *Fmr1^KO^* mice are the most widely employed experimental model for the study of FXS. The mice used here were in the C57BL/6J background, which is the most widely used inbred strain for biomedical research. The dietary exposure occurred over the entire lifespan as dams were treated pre-conception. We compared a wide range of outcome measures as a function diet, *Fmr1* genotype and sex. The major findings are that C57B/L6J mice grow faster when fed soy protein isolate with more pronounced effects in males than females and in *Fmr1^KO^* than WT. Increased growth in response to soy protein isolate was associated with increased body fat in females and increased lean mass in males. There were also altered activity and blood-based amino acid levels as a function of genotype and soy protein isolate.

These findings concur with our prior research demonstrating increased weight gain in *Fmr1^KO^* mice fed Purina 5015 compared AIN-76A and in *Fmr1^KO^* mice fed food pellets made from powdered soy-based infant formula compared to cow milk-based infant formula [11]. There are a few studies testing specialty diets in *Fmr1* model mice [12,14,15,17,33,34,35,36,37], but to our knowledge, no one else has compared the effects of standard rodent diets or protein source on *Fmr1^KO^* phenotypes [11,25,48]. There are published data supporting our findings of increased weight gain in response to soy in WT animals. Others have tested casein and soy protein-based diets (18% protein) in gerbils and found 21–69% higher body weight with the soy diet [62]. C57BL/6JBomTac mice exhibit elevated body weight when fed a high-fat/high-protein soy versus casein diet [63]. Soy protein isolate (20%) versus casein-based AIN-93G did not increase weight gain in C57BL/6J mice with dosing commenced at 3 weeks of age and body weight measured at a single time point at the end of the study after 13 weeks of experimental feeding [64]. Similarly, with our study, there was not a statistically significant increase in body weight in WT mice in response to soy at 13 weeks of age. We measured our mice from P3-P224 and only observed excess weight gain in response to soy in WT males at 5 weeks of age (P32 and P39).

There are also published findings that are inconsistent with ours regarding the effect of soy protein on weight gain. In male Wistar rats, there is a 22% decrease in body weight in response to soy versus casein diets (10% protein; casein diet supplemented with DL-methionine) [65]. Neonatal pigs fed soy (10% protein)-versus casein (8% protein)-based infant formulas have increased bone mineral density but not body weight [66], and pigs fed soy protein isolate versus a casein-based diet (50% protein restricted diet) exhibit decreased body weight [67]. These conflicting data that find decreased body weight in response to soy protein are in the context of suboptimal protein levels in the feeds and animal models other than mouse. In WT C57BL/6J, we found that mice fed soy-based infant formula formulated into pellets exhibit increased body weight compared to mice fed casein-based infant formula, where both infant formulas contain suboptimal protein and higher fat than recommended for rodents [11]. Thus, there may be species-specific effects in weight gain as a function of soy.

The timing of soy exposure is also expected to impact weight gain, but again there are contradictory findings. Pregnant Wistar rats fed soy versus casein-based AIN93G diets from day 3 of gestation throughout lactation followed by maintenance of pups on the casein-based diet for 15 weeks postweaning, exhibit increased food intake, body weight and fat pad mass in adult male animals [68]. On the other hand, monkeys born to dams consuming a typical American diet (TAD) derived from soy have similar body weights at birth but over a two-year period weigh significantly less [69]. Male CD1 mice fed a high soy isoflavone diet from conception exhibit reduced body weight and whole-body fat [70]. Maternal protein restriction during the preimplantation stage of development reduces *Fmr1* mRNA expression in the cortex [26]. It remains to be determined how proteins with reduced biological value like soy affect *Fmr1* expression.

We did not expect, and did not find, significant differences in bone density as a function of the *Fmr1* genotype. FMRP is expressed at significantly lower levels in skeletal muscle compared to other tissue in young and adult animals [71], and others find no genotype-specific differences in BMD [72]. Leboucher and colleagues observe increased body weight, muscle mass, and skeletal length and volume as well as reduced adiposity in male *Fmr1^KO^* compared to WT mice by X-ray tomography analysis at 4 months of age [72]. The only genotype-specific difference we observed by DEXA was decreased body fat in *Fmr1^KO^* versus *Fmr1^HET^* females (8 months old) when fed the casein diet. A major difference in the animal husbandry between the two studies was the feed; Leboucher and colleagues used 4RF25 Mucedola with the main ingredients of wheat and soybean meal. Interestingly, we found several diet-specific differences by DEXA in response to soy versus casein-based diets including increased lean mass, BMC and BMD in 8-month-old *Fmr1^KO^* males; increased body fat and total area in 8-month-old *Fmr1^KO^* females; increased bone area in *Fmr1^HET^* females; increased lean mass and TTM in 4-month-old WT males; and increased TTM in 4-month-old *Fmr1^HET^* females. These data indicate that soy is associated with altered growth metrics in mice and suggest that the choice of rodent diet may mask or exacerbate genotype-specific differences.

We expected and found greater BMD in male mice compared to females and in response to the soy diet. Soy protein increases BMD in C57BL/6 mice [73]. Conversely, soy contains oxalates, which can reduce bone density by preventing the uptake of calcium by bones. Collagen is the major protein constituent of bone mass. Connective tissue conditions such as hyperextensibility of finger joints, flat feet, increased skin elasticity, highly arched palates, mitral valve prolapse and aortic dilation are observed in many patients with FXS and suggest underlying abnormalities in collagen reminiscent of cutis laxa and Ehlers–Danlos syndromes [74,75,76]. Collagen constitutes 12% and 17%, respectively, of female and male mouse total body protein with the lowest levels in brain and liver (0.1%) and the highest in skin (20–40%), bones (25–35%) and tendons (40–50%) [77]. Interestingly, our concurrent mass spectrometry biomarker analyses identified collagen type 1 alpha 1 (COL1A1) [54].

There is conflicting data on activity levels in *Fmr1^KO^* mice with 24-7 monitoring of mice in the C57BL/6J background where others observe that *Fmr1^KO^* exhibit increased (6–8% during the light cycle, [78]), decreased (17–33% during the dark cycle, [79,80]), or no change in overall activity levels compared to WT mice [81]. We did not observe altered overall 24 h activity levels in *Fmr1^KO^* versus WT littermate mice in the C57BL/6J background; however, *Fmr1^KO^* male mice exhibit significantly increased activity during the beginning of the light cycle [15], which is reproduced here and concurs with the human FXS phenotype of having trouble falling asleep as mice are nocturnal. We also observe sex-specific differences with a shift in the time of acrophase in *Fmr1^HET^* and *Fmr1^KO^* females, and a more pronounced activity trough 3 h post-acrophase in *Fmr1^KO^* males, in response to soy. The effect of the soy diet on sleep states remains to be determined. Similar to others [80], we did not find altered habituation to the novel chambers, except for one time point with faster habituation in WT versus *Fmr1^KO^* males fed casein at the beginning of hour 2 post entry into the actigraphy chambers. We also did not observe a difference in circadian period. Our circadian period data averaged 24 h in both WT and *Fmr1^KO^* mice in agreement with one report [79] and versus another report observing 23.64 h (WT) and 24.67 h (*Fmr1^KO^*) [80].

### 4.2. Possible Mechanisms Underlying Dietary Soy-Induced Effects

Possible mechanisms underlying soy-induced effects on weight gain, bone density and activity levels in mice include altered hypothalamic function and amino acid balance as well as endocrine disrupting effects of phytoestrogens and opiate effects of soy peptides. The hypothalamus produces hormones involved in the regulation of body temperature, heart rate, blood pressure, appetite, mood, sleep, muscle and bone growth as well as the release of hormones from other glands. Increased body weight in *Fmr1^KO^* mice is associated with hypothalamic dysfunction with higher inhibitory GABAergic synaptic inputs in pro-opiomelanocortin (POMC) neurons [82]. There is a regulatory nexus involving microtubule-associated protein 1B (MAP1B), FMRP and agouti-related peptide (AgRP) in hypothalamic cells that is linked with food intake and body weight [83]. It remains to be determined if/how dietary soy protein affects hypothalamic function in *Fmr1^KO^* mice.

Regarding amino acid balance, essential amino acids (EAA) in the right proportions are required in the diet for adequate growth and maintenance of metabolically active tissues [84]. A balanced protein has high biological value and provides amino acids in roughly the proportion required by the body whereas a protein that is low in one or more EAA is defined as unbalanced with reduced biological value. There are large differences in EAA content between protein sources with lower levels in wheat-based versus intermediate levels in soy-based and higher levels in animal-based protein isolates such as casein [85]. Soy is an unbalanced protein with low levels of methionine. Our fasting blood plasma analyses indicated statistically decreased methionine levels in *Fmr1^KO^* female mice fed soy and a trend for decreased methionine in WT males fed soy (*p* = 0.067). We also observed decreased threonine levels in blood plasma from *Fmr1^KO^* male and female and WT male mice fed the soy diet here as well as in WT male and female mice fed chow (Teklad 2019 or Purina 5015) versus a purified ingredient diet (AIN-76A) [48]. Threonine-devoid diets are associated with an increased susceptibility to seizures in rats and Mongolian gerbils [86]. Plasma threonine concentration correlates with cortex and brainstem levels, which correlate with glycine concentrations in these brain regions that could affect the neurotransmitters balance in the brain [87]. Amino acid levels in the *Fmr1^KO^* mouse brain in response to diet remain to be determined.

Serum levels of branched chain amino acids (BCAA: leucine, isoleucine, valine) are lower in children with ASD [88]. Milk contains a high level of leucine, which stimulates muscle protein synthesis and inhibits protein degradation. We did not observe altered levels of leucine as a function of the *Fmr1* genotype, sex or diet. Isoleucine levels were lower in *Fmr1^HET^* mice fed soy compared to the casein diet. Valine levels were lower in WT male mice fed soy compared to the casein diet. Total BCAA were not different dependent on genotype or diet. Excitatory amino acids, aspartate and glutamate, are significantly elevated in children with ASD [89]. D-aspartate modulates NMDAR and mGluR_5_/mTOR/4E-BP signaling and rescues cognitive and locomotor coordination deficits in *Fmr1^KO^* mice [90]. We did not observe differences in levels of plasma excitatory amino acids dependent on casein or soy-based diets.

Others have conducted metabolomic screens in neurodevelopmental models. Davidovic and colleagues identified metabolomic signatures associated with *Fmr1^KO^* mouse brain regions (FVB background) [91]; Gruss and Braun measured metabolite levels as a function of *Fmr1^KO^* brain region and age (FVB/NJ background) [92,93]; Shi and colleagues quantitated metabolites in hippocampus of *Fmr1^KO^* mice (C57BL/6J background) [94]; Menzies and colleagues measured metabolite levels in *Fmr1^KO^* mouse plasma (FVB/129 background) [95]; and Smith and colleagues identified metabolomic signatures associated with autism blood plasma [96,97]. The mouse brain studies concur in finding increased taurine in *Fmr1^KO^* brain. Compared to sex-matched WT littermates, *Fmr1^KO^* male and female mouse plasma have elevated levels of glutamine and asparagine; *Fmr1^KO^* males also have elevated glutamate, aspartate and proline; and *Fmr1^KO^* females have lower levels of glutamate and aspartate compared to *Fmr1^KO^* males [95]. Of interest, Davidovic and colleagues found reduced levels of the excitatory amino acids aspartate (cerebellum) and glutamate (cortex) in the brains of *Fmr1^KO^* male mice versus wild type littermates whereas Menzies and colleagues found elevated levels in plasma. We found reduced glutamine in *Fmr1^KO^* male mouse plasma compared to WT on the casein diet; we did not observe elevated glutamate, aspartate or proline in males on either casein or soy diets; and we did not observe reduced glutamate and aspartate in *Fmr1^KO^* females versus males on either diet. It remains to be determined how chows versus purified ingredient diets affect metabolomic signatures in the brain and blood in FXS models and if levels correlate between species and tissues. Of note, the Davidovic mice were in the C57BL/6J background and maintained on a Mucedola 4RF21 chow and the Menzies’ mice were in the FVB/129 background and maintained on a Teklad chow, while both our casein and soy diets are AIN-93G-based and our mice were in the C57BL/6J background. When we compared blood-based biomarkers in WT mice maintained on Teklad 2019 chow versus a AIN-76A purified ingredient diet [48], we found elevated isoleucine, methionine, tyrosine, valine and BCAA in females maintained on AIN-76A as well as increased lysine and threonine in both females and males. Human studies find altered ratios of blood amino acids associated with autism; specifically, elevated glutamine, glycine and ornithine and reduced BCAA [96,97]. In WT mice, we found these same signatures in response to a Purina 5015 diet versus AIN-76A [48]. Here, we find an increased ratio of glycine to BCAA in male versus female *Fmr1^KO^* mice, as well an increased ratio of glycine to isoleucine, when fed the soy diet. We find a reduced ratio of ornithine to BCAA in *Fmr1^KO^* female mice fed soy versus casein. 

Phytoestrogens are another possible mechanism underlying soy protein isolate-induced effects on growth and activity levels in mice. Soy protein is rich in a type of phytoestrogen called isoflavones, which are polyphenols found in legumes. Commercial rodent diets formulated with soy protein deliver large daily doses of bioavailable isoflavones to the animals reaching high steady state serum concentrations in mice at 2238 ± 531 ng/mL, which exceeds endogenous estrogen levels by 30,000–60,000-fold [61]. These isoflavones can interfere with the normal function of endogenous estrogen. It is interesting that the timing of excess weight gain in response to soy in female mice occurs at P9–P18 in *Fmr1^HET^* and at P6–P18 and P168–P224 in *Fmr1^KO^*, which is prior to puberty onset in C57BL/6J (females P28–P36) [98,99,100] and at the cessation of reproductive function in *Fmr1^KO^* females (average age of last litter, FVB strain, P163) [101]. In WT males, the timing of excess weight gain was P32–P39, which is immediately after puberty onset in C57BL/6J (P25–P31) [98]. We find increased body weight in male *Fmr1^KO^* from postnatal day 6 (P6) through P224. It remains to be determined how the lack of FMRP affects gonadal hormone levels in *Fmr1^KO^* mice over their lifespan and in response to diet and sex. Others have shown early vaginal opening; early cessation of reproductive function; no difference in body weight at any age; and elevated luteinizing hormone (LH), follicle-stimulating hormone (FSH), testosterone and progesterone at diestrus in FVB *Fmr1^KO^* females compared to WT controls [101]. Overall, these data suggest an interaction between reproductive hormones and weight gain in mice, and that the *Fmr1^KO^* mutation, particularly in males, could be more sensitive to the endocrine disrupting effects of soy phytoestrogens.

The most abundant phytoestrogen in mouse plasma was R,S-equol-7-O-β-D-glucuronide, which averaged 100.5 ng/mL (228 nM) in WT male mice and 18.25 ng/mL (41.4 nM) in *Fmr1^HET^* female mice which is consistent with the literature that levels are higher in male (932 ± 300 nM) versus female (356 ± 64 nM) mice fed a diet supplemented with soy extract [102]. Equol is metabolized from daidzein by the gut microbiota followed by phase II conjugation with glucuronic acid or sulfate [102,103]. It is considered the most biologically active metabolite in terms of estrogenicity [102,103]. S-(−)equol binds preferentially to estrogen receptor beta (ERβ) and antagonizes dihydrotestosterone [104]. In contrast to humans, all mice are equol producers [102,103]. We did not observe an *Fmr1* genotype-specific difference in any of the plasma isoflavones tested here. It remains to be determined if there are altered levels of estrogen or androgen hormones, hormone receptors or intestinal equol-producing bacteria as a function of the *Fmr1* genotype and soy diet.

We assessed two gonadal anatomical measures in the mice as a function of genotype and diet. The only statistically significant differences observed were increased AGD in *Fmr1^KO^* males and testes volume in WT males in the soy-fed versus Purina 5015 cohorts, where both diets contain soy protein. There was a trend for increased testes volume in WT males fed soy compared to casein. Others have shown that perinatal exposure of male rats to genistein causes temporary, prepubertal urogenital abnormalities with smaller AGD and testis size as well as delayed preputial separation [105]. Feeding marmosets soy infant formula increases testis weight 14% at 120–138 weeks of age [106], but there is no difference at 35–45 days old [107]. The National Institute of Environmental Health Sciences Infant Feeding and Early Development (IFED) Study of a prospective cohort of maternal-infant dyads exclusively fed soy formula, cow milk formula, or breast milk did not find statistical differences in trajectories of hormone concentrations or anatomical measures between boys fed soy formula and cow milk formula; however, compared with breastfed, soy formula-fed boys had a more rapid increase in penile length and slower initial lengthening of AGD with data adjusted for weight-for-length z-scores [35,36,108]. Despite lack of statistical significance with *N* = 38–55 infants per cohort, the trajectories show trends for the highest penile length in the soy cohort followed by cow milk formula and then breast milk. The AGD trajectories overlapped for cow milk formula and breast milk, but the trend was lower with soy up to about 120 days of age and higher with soy thereafter. In male piglets, consumption of soy formula caused a small increase in absolute testes weight relative to sow milk and cow milk formula groups that was lost when corrected for body weight [109]. Our findings of increased AGD in *Fmr1^KO^*, and testes volume in WT mice, in response to soy versus Purina 5015 suggests that dietary components in addition to soy contribute to altered gonadal phenotypes.

A final possible mechanism that we will discuss here is opioid-like peptides. Proteins can be digested to produce gluten exorphins (wheat), casomorphins (milk) and soymorphins (soybean), which have opioid activity [110,111]. Soymorphins are ligands for m-opioid receptors [112], and affect intestinal transit, hormone secretions and mucus levels in mice [110,113]. It is known that FXS is associated with increased GI problems [114], and the use of soy-based infant formula is associated with more severe GI problems [52]. The effects of opioid-like peptides in the context of FXS remain to be determined. The kappa opioid and sigma-1 non-opioid receptors are under investigation as therapeutic targets for FXS [115,116,117].

Possible molecular mechanisms underlying soy-induced effects in *Fmr1^KO^* mice have been previously described [24,38].

### 4.3. Abnormal Pathology in Response to Purified Ingredient Casein Protein-Based Diet

Regarding the observation of steatosis (too much fat in the liver) in mice fed the casein diet, steatosis is indicative of mitochondrial dysfunction. Mitochondrial dysfunction is evident in *Fmr1^KO^* mice [118]; however, we observed a high incidence of steatosis in all strains fed the casein diet (30% *Fmr1^HET^* female, 40% *Fmr1^KO^* female, 25% WT male, and 31% *Fmr1^KO^* male). We did not observe any steatosis with the matched soy diet and low levels with the chows. Expression of an expanded repeat (90 CGG) RNA causes liver pathology with mild steatosis in a C57BL/6 fragile X-associated tremor/ataxia syndrome (FXTAS) mouse model where the liver is pink in color and pale compared to the typical dark-reddish brown color [119]. Purified ingredient diets formulated with high fat or fructose, or deficient in methionine and choline, are associated with liver steatosis [120,121]; however, the diets used here contain American Institute of Nutrition (AIN) recommended levels of macro- and micronutrients for mice and 10% sucrose as the sugar source. There is a report of liver steatosis in Wistar rats fed an AIN-93 diet [122]. The high incidence of steatosis in all mouse cohorts fed the casein-based diet and the low incidence in the matched soy-based purified ingredient diet in this study, suggest that casein is contributing to liver disease irrespective of FMRP levels. It remains to be determined in larger cohorts of mice if liver disease is worse with FMRP deficiency. Steatosis can be caused by impaired very low density lipoprotein (VLDL) secretion, fatty acid uptake and triglyceride synthesis [123]. Others have observed reduced triglycerides, total cholesterol, carnitine, leptin and insulin as well as increased fatty acids and ketone bodies in *Fmr1^KO^* mice [124]. The 5′-UTR of the mRNAs coding for the FMRP and VLDL receptor (VLDLR) both contain a polymorphic CGG triplet repeat [125,126], and the mRNA for VLDLR is an FMRP-binding target [127]. Thus, multiple molecular pathways related to FMRP could contribute to a fatty liver. Overall, there are varied adverse health events associated with multiple single-source diets in *Fmr1*-mutant mice: chows (*Fmr1^KO^*, seizures), soy/purified (*Fmr1^KO^*, increased weight gain), and casein/purified (steatosis).

### 4.4. Study Limitations

The major translational limitation of this work is that rodents and humans metabolize phytoestrogens differently. Regardless, the findings have important implications for biomedical research because drugs are tested in rodents before they progress to clinical trials. Another limitation is that the study was conducted in a single strain of mice, C57BL/6J mice. There is evidence from the literature that *Fmr1^KO^* male and female mice (Jackson Laboratory stock #004624) in a mixed FVB/129 background weigh more than WT littermate controls, with higher lean mass for both sexes and increased fat mass for females [95]. We are currently testing *Fmr1^KO^* mice in the FVB strain to test replicability of the findings as a function of the soy diet. A limitation with all research conducted in the *Fmr1^KO^* mouse model is that these mice have the *Fmr1* gene knocked out with a neomycin cassette [128], whereas the mutation in the human disorder is a trinucleotide repeat expansion. The significance of toxic functions associated with mutant RNA from an *Fmr1* trinucleotide repeat expansion versus loss of FMRP remains to be determined. Regardless, in both rodents and humans, loss of FMRP expression is associated with disease phenotypes [128,129]. There is a potential source of bias as investigators were not blind to diet treatment. The diets were color-coded to facilitate chronic dosing over an 8-month period.

### 4.5. Clinical Applications

While obesity is a relative term mostly used to describe human studies, a 48% increase in abdominal adipose tissue, in conjunction with a total body weight that exceeds one standard deviation from the Jackson Laboratory mean, could be construed as obesity in *Fmr1^KO^* male mice in response to the purified ingredient soy diet. Considering the prevalence of obesity in FXS and that 25% of infants with FXS are fed soy-based infant formula [52], which has the potential to affect epigenetic programming, our findings warrant further investigation of the long-term effects of single-source soy-based diets on FXS phenotypes in both rodents and humans. There are alternative feeding options to soy-based infant formula for infants who cannot breastfeed or tolerate cow milk-based infant formulas.

## 5. Conclusions

In summary, dietary consumption of soy protein isolate by C57BL/6J mice causes increased growth, which can be attributed to increased lean mass in males and fat mass in females. Findings are more pronounced in *Fmr1^KO^* versus WT and in males versus females. Testes volume is the only established *Fmr1^KO^* phenotype tested here that persists irrespective of diet. These data confirm our hypothesis that soy is associated with increased weight gain in *Fmr1^KO^* mice. These findings could have important implications for FXS and other neurodevelopmental disorders, which are highly comorbid with obesity. The generalizability of the findings between species and how genetics affects the metabolism of phytoestrogens as well as underlying mechanisms remain to be determined with future research. The findings are clinically and translationally relevant to humans for two reasons: mice are routinely used for preclinical drug testing and diet could confound the results, and the equivalent of feeding laboratory animals single-source, soy-based chows is feeding babies soy-based infant formulas. If postnatal consumption of single-source diets such as soy protein-based infant formula is contributing to the current pediatric autism and obesity epidemics, particularly in association with autism-related genes, infant nutrition policy needs to be reevaluated and revised. In the United States, the National Toxicology Program Center for the Evaluation of Risks to Human Reproduction states that there is minimal concern of adverse effects on development for infants consuming soy-based infant formula [130], whereas The European Academy of Allergy and Clinical Immunology (EAACI) recommends against the use of soy-based infant formula during the first 6 months of life as a means to prevent food allergy [131]. Regardless of generalizability of our findings to the human diet, the data speak to the importance of increased awareness during study design regarding potential dietary effects on outcome measures and the need for rigor in reporting diet details in biomedical rodent research.

## Figures and Tables

**Table 1:** Rodent and human phenotypes in response to soy-based diets.

Phenotype	Rodent	Human
Seizures	↑ [25]	↑ [50,53]
Body Weight	↑ [11,48], [Figures 1 and 2]	↔ [11,51]
Hyperactivity, ADHD	↑ [Figure 3]	↑ [51,53]; ↔ [49]
Autism	ND	↑ [23,52]
RRSB ^1^	ND	↑ [49]
Motor Coordination	↑ [Figure 4]	↔ [23]
Learning	↑ [11]; ↔ [Figure 5]	↓ [23]
GI Problems	ND	↑ [52]
Allergies	ND	↑ [51,52]
Altered Blood Biomarkers	Yes [11], [Figures 6 and 7]	ND
Organ Weight	↑ [Figure 8]	ND

^1^ Restricted, repetitive and stereotyped behavior (RRSB).

**Table 2:** Number of animals per experiment.

Figure	Casein Diet (*n*)	Soy Diet (*n*)
Figure 2A	27 *Fmr1^HET^* female, 36 *Fmr1^KO^* female	40 *Fmr1^HET^* female, 43 *Fmr1^KO^* female
Figure 2B	23 *Fmr1^HET^* female, 31 *Fmr1^KO^* female	23 *Fmr1^HET^* female, 39 *Fmr1^KO^* female
Figure 2C	30 WT male, 38 *Fmr1^KO^* male	49 WT male, 48 *Fmr1^KO^* male
Figure 2D	28 WT male, 33 *Fmr1^KO^* male	48 WT male, 45 *Fmr1^KO^* male
Figure 3	14 *Fmr1^HET^* female, 12 *Fmr1^KO^* female 10 WT male, 16 *Fmr1^KO^* male	16 *Fmr1^HET^* female, 16 *Fmr1^KO^* female 20 WT male, 19 *Fmr1^KO^* male
Figure 4A	16 *Fmr1^HET^* female, 17 *Fmr1^KO^* female	25 *Fmr1^HET^* female, 28 *Fmr1^KO^* female
Figure 4B	14 WT male, 20 *Fmr1^KO^* male	32 WT male, 28 *Fmr1^KO^* male
Figure 5A	16 *Fmr1^HET^* female, 17 *Fmr1^KO^* female	25 *Fmr1^HET^* female, 28 *Fmr1^KO^* female
Figure 5B	14 WT male, 20 *Fmr1^KO^* male	32 WT male, 28 *Fmr1^KO^* male
Figure 6	10 *Fmr1^HET^* female, 10 *Fmr1^KO^* female 8 WT male, 13 *Fmr1^KO^* male	18 *Fmr1^HET^* female, 21 *Fmr1^KO^* female 21 WT male, 20 *Fmr1^KO^* male
Figure 7	5 *Fmr1^HET^* female, 8 *Fmr1^KO^* female 4 WT male, 9 *Fmr1^KO^* male	9 *Fmr1^HET^* female, 8 *Fmr1^KO^* female 11 WT male, 8 *Fmr1^KO^* male
Figure 8	10 *Fmr1^HET^* female, 10 *Fmr1^KO^* female 8 WT male, 13 *Fmr1^KO^* male	19 *Fmr1^HET^* female, 22 *Fmr1^KO^* female 21 WT male, 20 *Fmr1^KO^* male
Figure 9	6 *Fmr1^HET^* female, 7 *Fmr1^KO^* female 6 WT male, 7 *Fmr1^KO^* male	6 *Fmr1^HET^* female, 6 *Fmr1^KO^* female 11 WT male, 8 *Fmr1^KO^* male
Figure 10	9 *Fmr1^HET^* female, 16 *Fmr1^KO^* female 16 WT male, 16 *Fmr1^KO^* male	12 *Fmr1^HET^* female, 21 *Fmr1^KO^* female 14 WT male, 12 *Fmr1^KO^* male

## Data Availability

Data are contained within the article or Supplementary Materials.

## Supplementary Materials

The following supporting information can be downloaded at: https://www.mdpi.com/article/10.3390/nu16020284/s1, Figure S1: Phytoestrogen levels in diets; Figure S2: Percent change in body weight as a function of soy protein isolate; Figure S3: Rest-activity rhythms in adult mice; Figure S4: Motor coordination in response to sex, Fmr1 genotype and soy protein isolate; Figure S5: Learning and memory in response sex, Fmr1 genotype and soy protein isolate; Figure S6: Blood-based amino acid levels in response to sex, Fmr1 genotype and soy protein isolate; Figure S7: Blood-based phytoestrogen levels in response to sex, Fmr1 genotype and soy protein isolate; Figure S8: Glucose levels in response to sex, Fmr1 genotype and soy protein isolate; Figure S9: DEXA analysis in response to soy protein isolate in 8-month-old mice; Figure S10: DEXA analysis in response to soy protein isolate in 4-month-old mice; Table S1: Casein and soy-protein based matched diets; Table S2: Mass spectrometry conditions; Table S3: Abdominal disease as a function of genotype and diet; Table S4: Diseased liver as a function of genotype and diet; and Table S5: Abdominal disease as a function of diet.
