# Supplementary material for: Effects of Soy Protein Isolate on Fragile X Phenotypes in Mice
Source: Nutrients. 2024 Jan 18;16(2):284. doi: 10.3390/nu16020284 (PMC10819477; doi:10.3390/nu16020284)
Supplement: Supplementary file 1 [file nutrients-16-00284-s001.zip › nutrients-2771458-supplementary.pdf]

Supplementary Material

Effects of Soy Protein Isolate on Fragile X Phenotypes in Mice

Pamela R. Westmark [1,†], Greg Lyon [2,†], Alejandra Gutierrez [3,†], Brynne Boeck [4], Olivia Van Hammond [2], Nathan Ripp [4], Nicole Arianne Pagan-Torres [5], James Brower [6], Patrice K. Held [6], Cameron Scarlett [7] and Cara J. Westmark [8,*]

[1] Department of Neurology, University of Wisconsin, Madison, WI 53706, USA; prwestmark@wisc.edu (P.R.W.)
[2] Undergraduate Research Scholars Program, University of Wisconsin, Madison, WI 53706, USA; gregorylyon6@gmail.com (G.L.); ovanhammond@wisc.edu (O.V.H.)
[3] Molecular Environmental Toxicology Master's Program, University of Wisconsin, Madison, WI 53706, USA; gutierrez9@wisc.edu (A.G.)
[4] Neurology Undergraduate Research, University of Wisconsin, Madison, WI 53706, USA; bboeck@wisc.edu (B.B.); njripp2@wisc.edu (N.R.)
[5] Molecular Environmental Toxicology Summer Research Opportunities Program, University of Wisconsin, Madison, WI 53706, USA; nicole.pagantorres@bsd.uchicago.edu (N.P.T.)
[6] Wisconsin State Laboratory of Hygiene, University of Wisconsin, Madison, WI 53706, USA; james.brower@slh.wisc.edu (J.B.); patrice.k.held@oha.oregon.gov (P.K.H.)
[7] School of Pharmacy, University of Wisconsin, Madison, WI 53706, USA; cameron.scarlett@wisc.edu (C.S.)
[8] Department of Neurology and Molecular Environmental Toxicology Center, University of Wisconsin, Madison, WI 53706, USA
* Correspondence: westmark@wisc.edu (C.J.W.); Tel.: +1-(608)-262-9730
† These authors contributed equally to this work.

1A

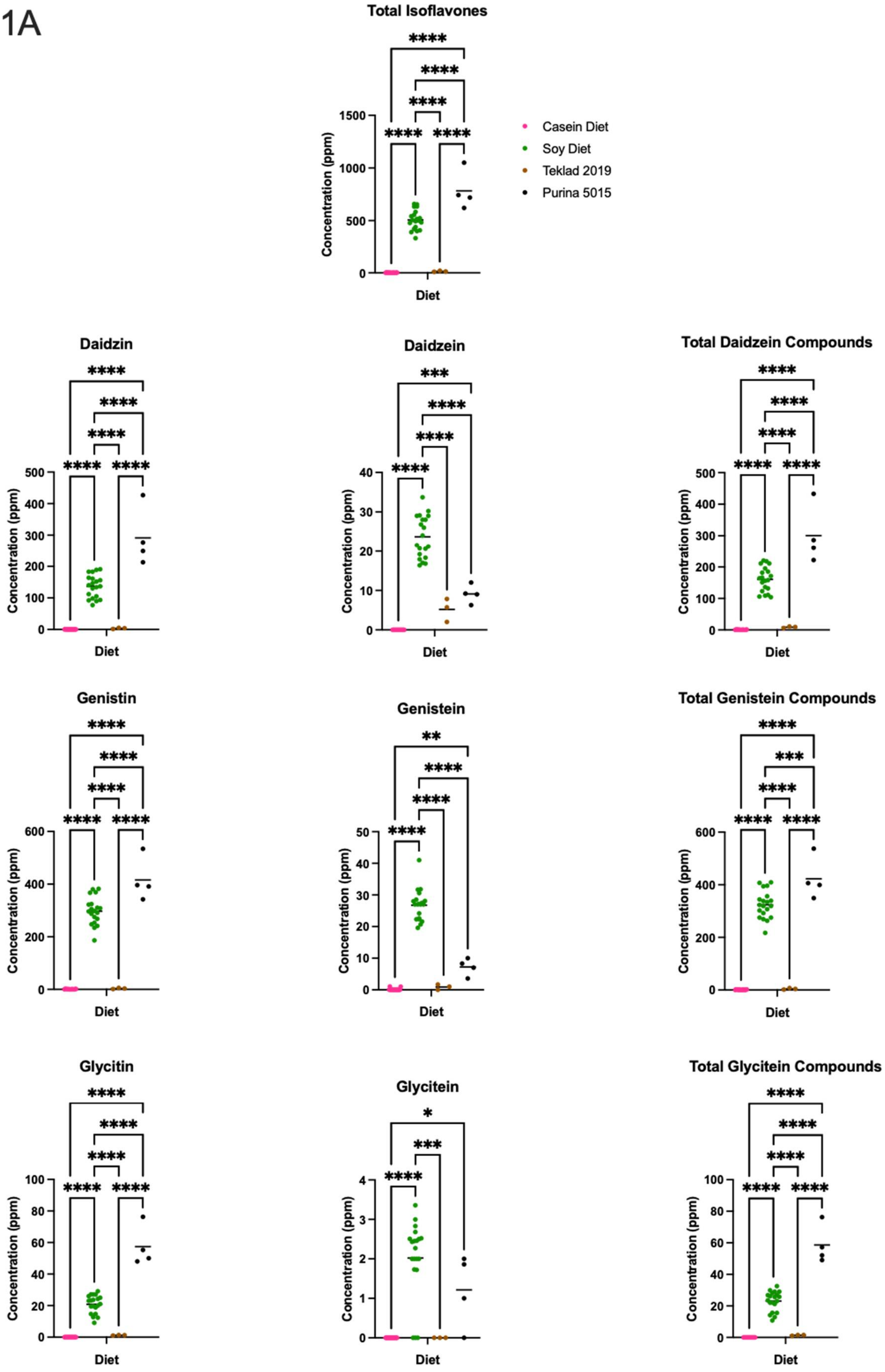
Total Isoflavones
Concentration (ppm)
Diet
Casein Diet
Soy Diet
Teklad 2019
Purina 5015
Daidzin
Daidzein
Total Daidzein Compounds
Genistin
Genistein
Total Genistein Compounds
Glycitin
Glycitein
Total Glycitein Compounds

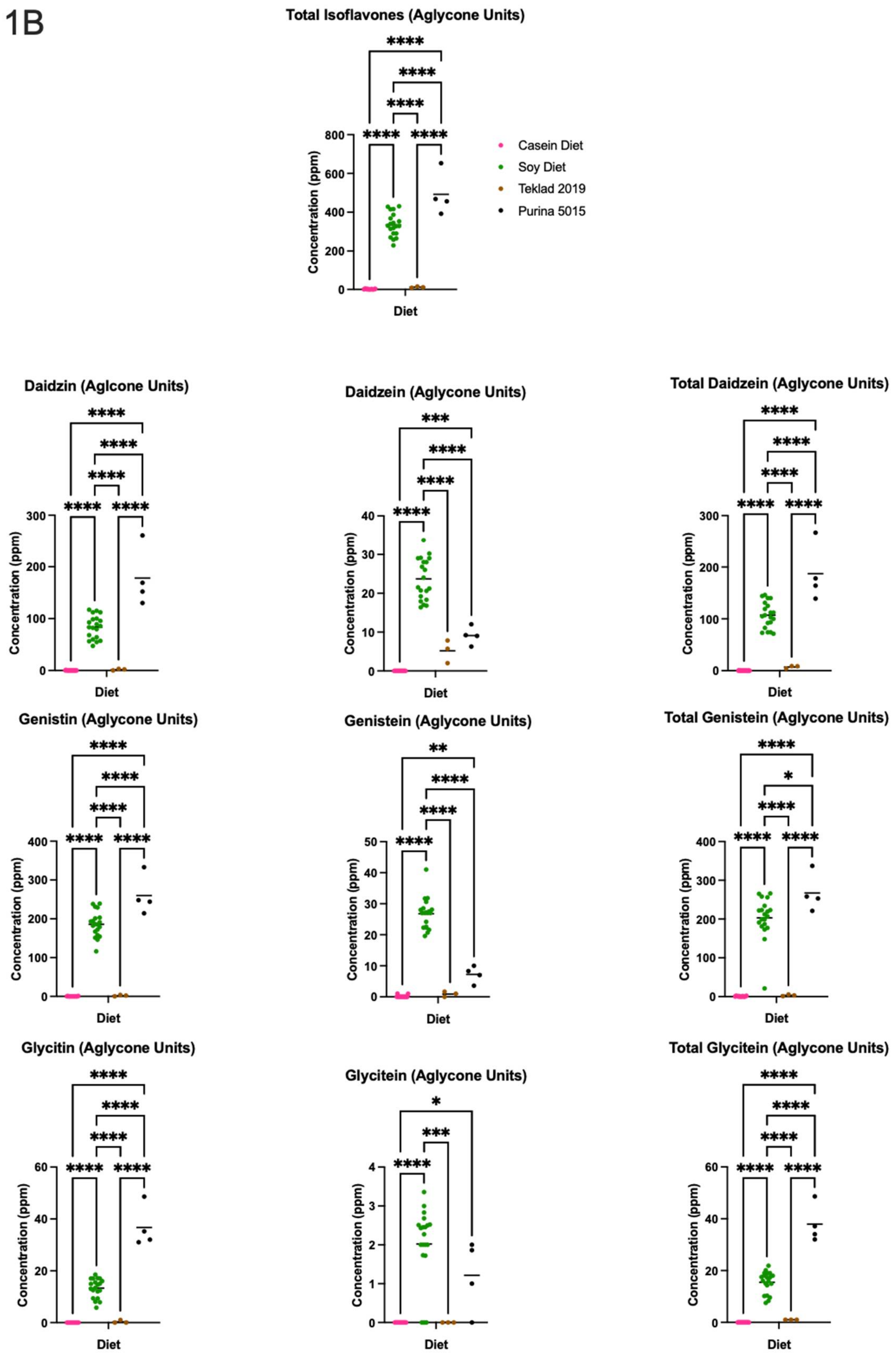

Figure S1. Phytoestrogen levels in diets. The levels of (A) daidzin, daidzein, genistin, genistein, glycitin, glycitein, and their (B) aglycone derivatives were determined in n=16 (casein, pink symbols) and n=20 (soy, green) batches of feed synthesized over a 38-month period and compared with n=3 batches of Teklad 2019 (brown) and n=4 batches of Purina 5015 (black).

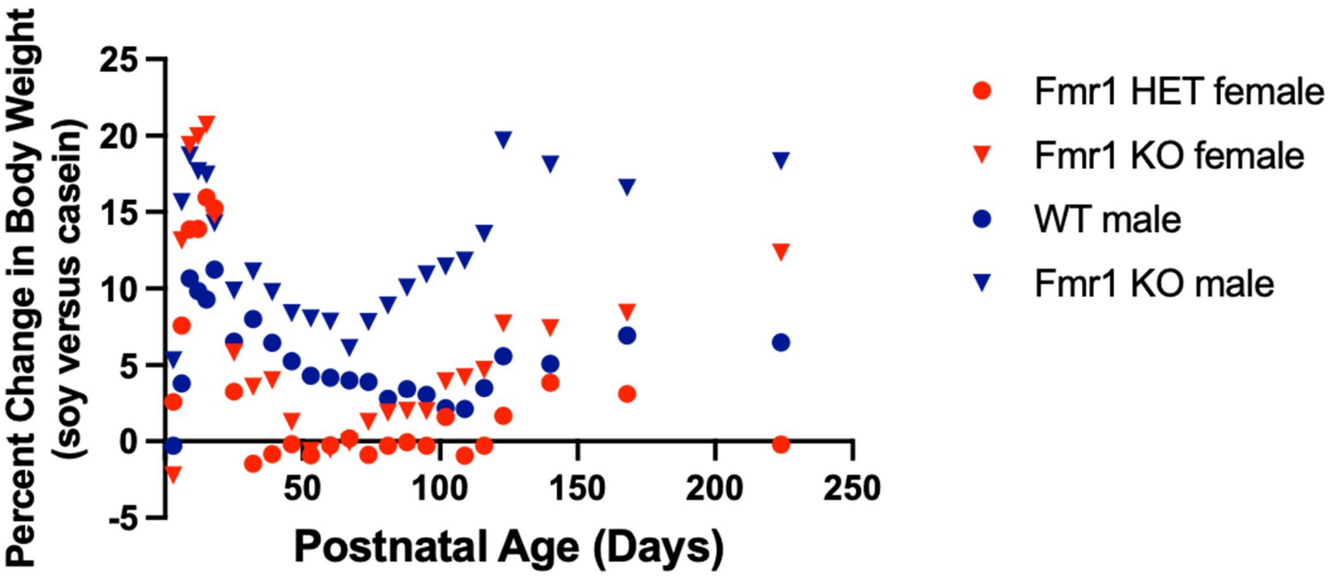

Figure S2. Percent change in body weight as a function of soy protein isolate. The data from Figure 2 are replotted as a percent change between casein and soy diets with females denoted (red), males (blue), WT or *Fmr1*^{HET} (circles) and *Fmr1*^{KO} (triangles).

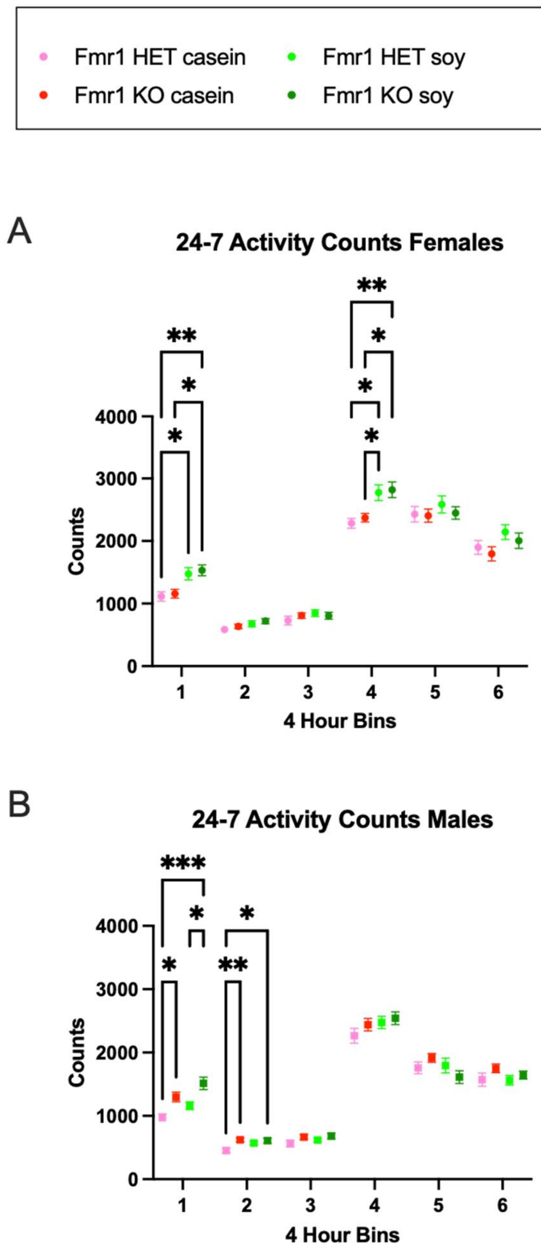

Figure S3. Rest-activity rhythms in adult mice. Each gross movement of the animal was recorded as an activity count for *Fmr1*^{HET} females fed casein diet (pink, n=14), *Fmr1*^{KO} females fed casein diet (red, n=12), *Fmr1*^{HET} females fed soy diet (light green, n=16), *Fmr1*^{KO} females fed soy diet (dark green, n=16), WT males fed casein diet (pink, n=10), *Fmr1*^{KO} males fed casein diet (red, n=16), WT males fed soy diet (light green, n=20), and *Fmr1*^{KO} males fed soy diet (dark green, n=19). Data were analyzed with an ANOVA 2-way model and Tukey's multiple comparison test. The 24-7 activity count data from Figure 3B are replotted in 4-hour increments for females: Bin x Genotype/Diet F (15, 270) = 2.358 p=0.0034; Bin F (3.613, 195.1) = 542.4, p<0.0001; Genotype/Diet F (3, 54) = 3.517, p=0.0211; Subject F (54, 270) = 5.520, p<0.0001; and Geisser-Greenhouse's epsilon = 0.7225. The 24-7 activity count data from Figure 3B are replotted in 4-hour increments for males: Bin x Genotype/Diet F (15, 305) = 3.122, p<0.0001; Bin F (3.470, 228.1) = 535.4, p<0.0001; Genotype/Diet F (3, 61) = 1.755, p=0.1653; Subject F (61, 305) = 5.185, p<0.0001; and Geisser-Greenhouse's epsilon = 0.7479.

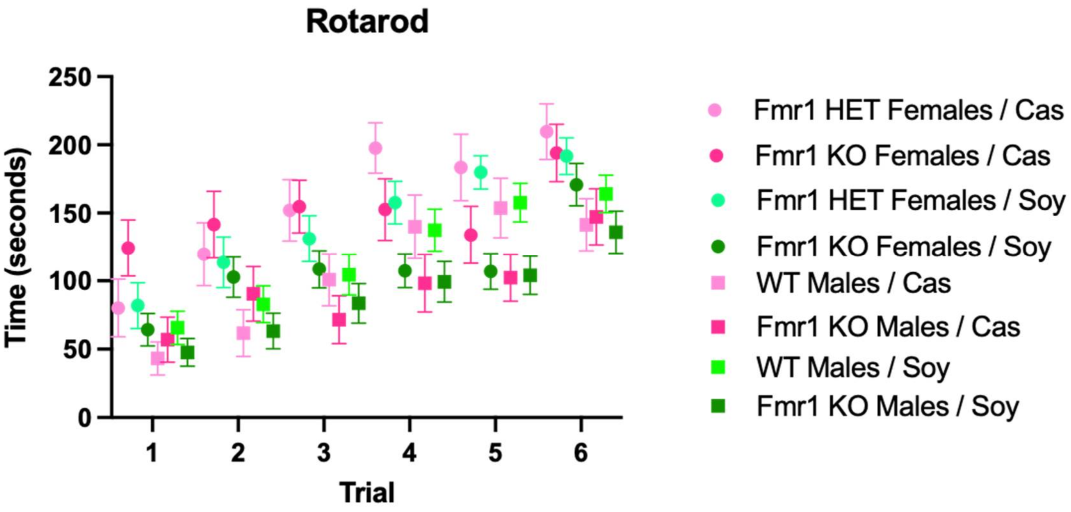

Figure S4. Motor coordination in response to sex, *Fmr1* genotype and soy protein isolate. The data from Figure 4 are replotted to include comparison of sex on the same graph with females denoted (circles) and males (squares). One-way ANOVA analyses for individual trials indicates statistically significant differences in trial 1 ($Fmr1^{KO}$ females/casein versus WT males/casein and $Fmr1^{KO}$ females/casein versus $Fmr1^{KO}$ males/soy), trial 2 ($Fmr1^{KO}$ females/casein versus $Fmr1^{KO}$ males/soy), trial 3 ($Fmr1^{KO}$ females/casein versus $Fmr1^{KO}$ males/casein), trial 4 ($Fmr1^{HET}$ females/casein versus $Fmr1^{KO}$ females/soy, $Fmr1^{HET}$ females/casein versus $Fmr1^{KO}$ males/casein, $Fmr1^{HET}$ females/casein versus $Fmr1^{KO}$ males/soy), trial 5 ($Fmr1^{HET}$ females/casein versus $Fmr1^{KO}$ females soy, $Fmr1^{HET}$ females/casein versus $Fmr1^{KO}$ males/casein, $Fmr1^{HET}$ females/casein versus $Fmr1^{KO}$ males soy, $Fmr1^{HET}$ females/soy versus $Fmr1^{KO}$ female/soy, $Fmr1^{HET}$ females/soy versus $Fmr1^{KO}$ males/casein, $Fmr1^{HET}$ females/soy versus $Fmr1^{KO}$ males /soy), and trial 6 (none).

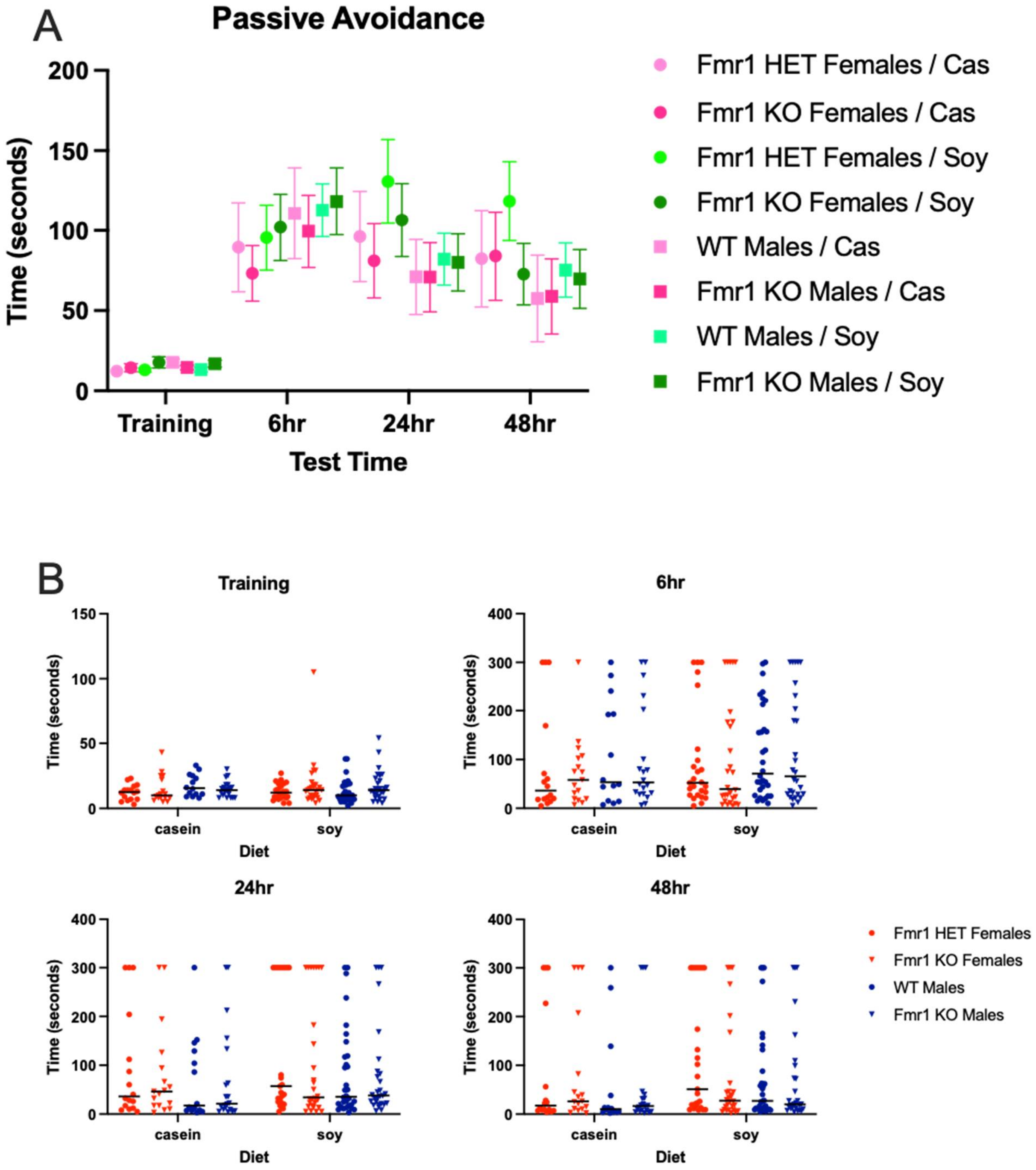

Figure S5. Learning and memory in response sex, *Fmr1* genotype and soy protein isolate. The data from Figure 5 are replotted to: (A) include comparison of sex on the same graph with females denoted (circles) and males (squares); and (B) to show the spread in the raw data for training, 6 hr, 24 hr and 48 hr latency times with females denoted (red), males (blue), WT or $Fmr1^{HET}$ (circles) and $Fmr1^{KO}$ (triangles).

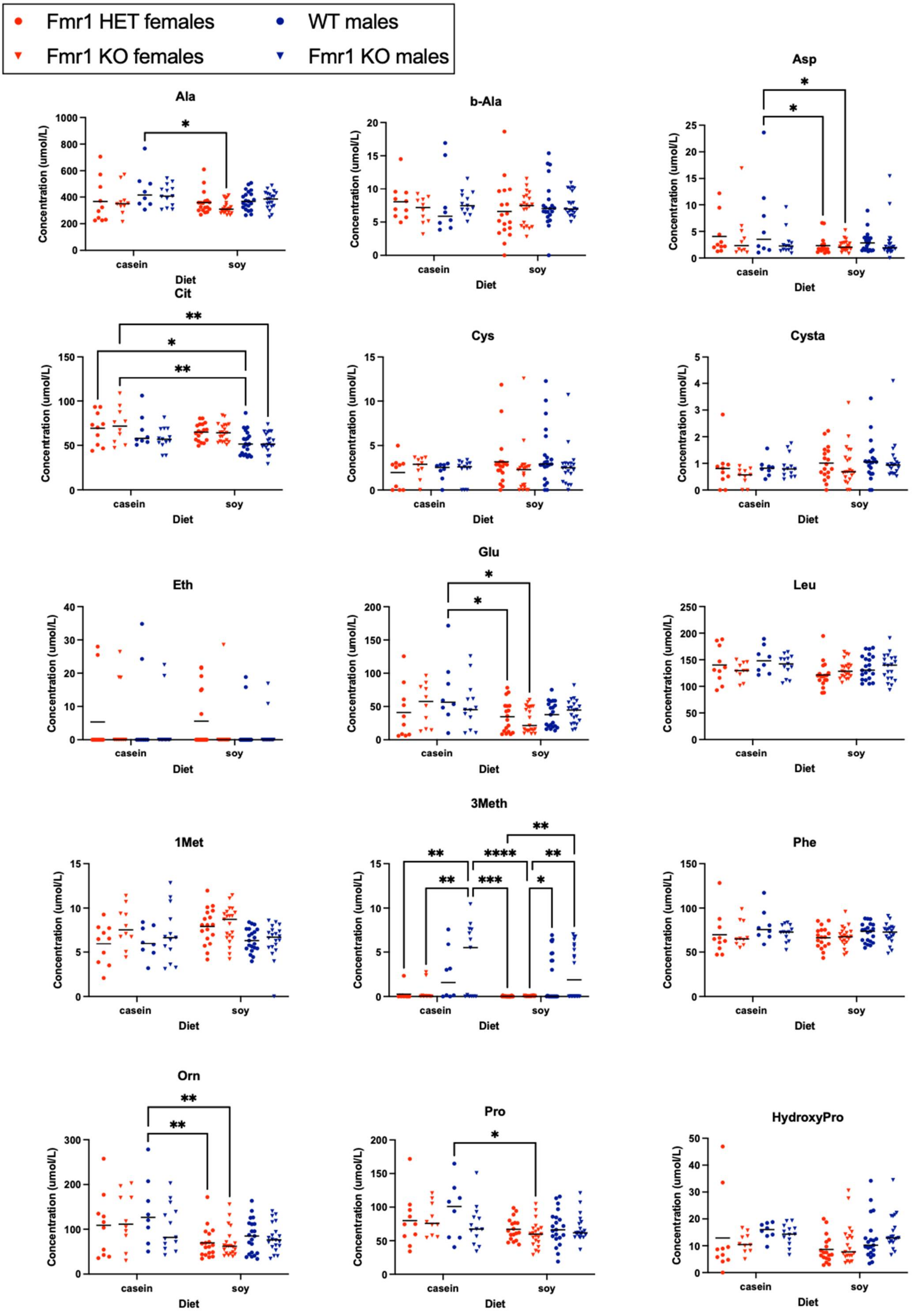
Fmr1 HET females
Fmr1 KO females
WT males
Fmr1 KO males
Ala
b-Ala
Asp
Cit
Cys
Cysta
Eth
Glu
Leu
1Met
3Meth
Phe
Orn
Pro
HydroxyPro
Concentration (umol/L)
casein
soy
Diet

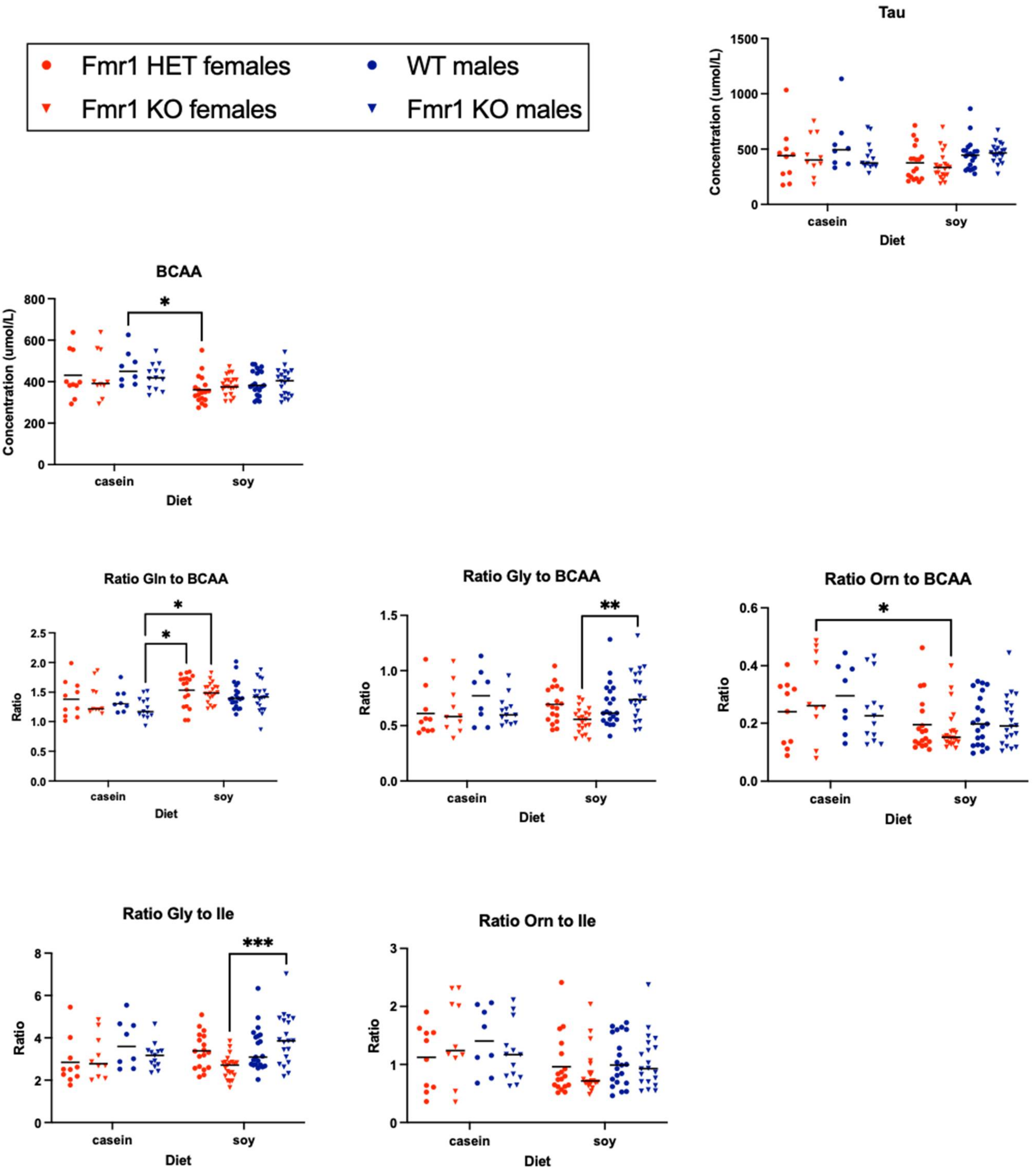

Figure S6. Blood-based amino acid levels in response to sex, *Fmr1* genotype and soy protein isolate. Blood amino acid levels were quantitated as a function of genotype and diet. Data were analyzed with an ANOVA 2-way model and Tukey's multiple comparison test. Amino acids, BCAA and ratios of specific amino acids to BCAA not presented in Figure 6 are provided here.

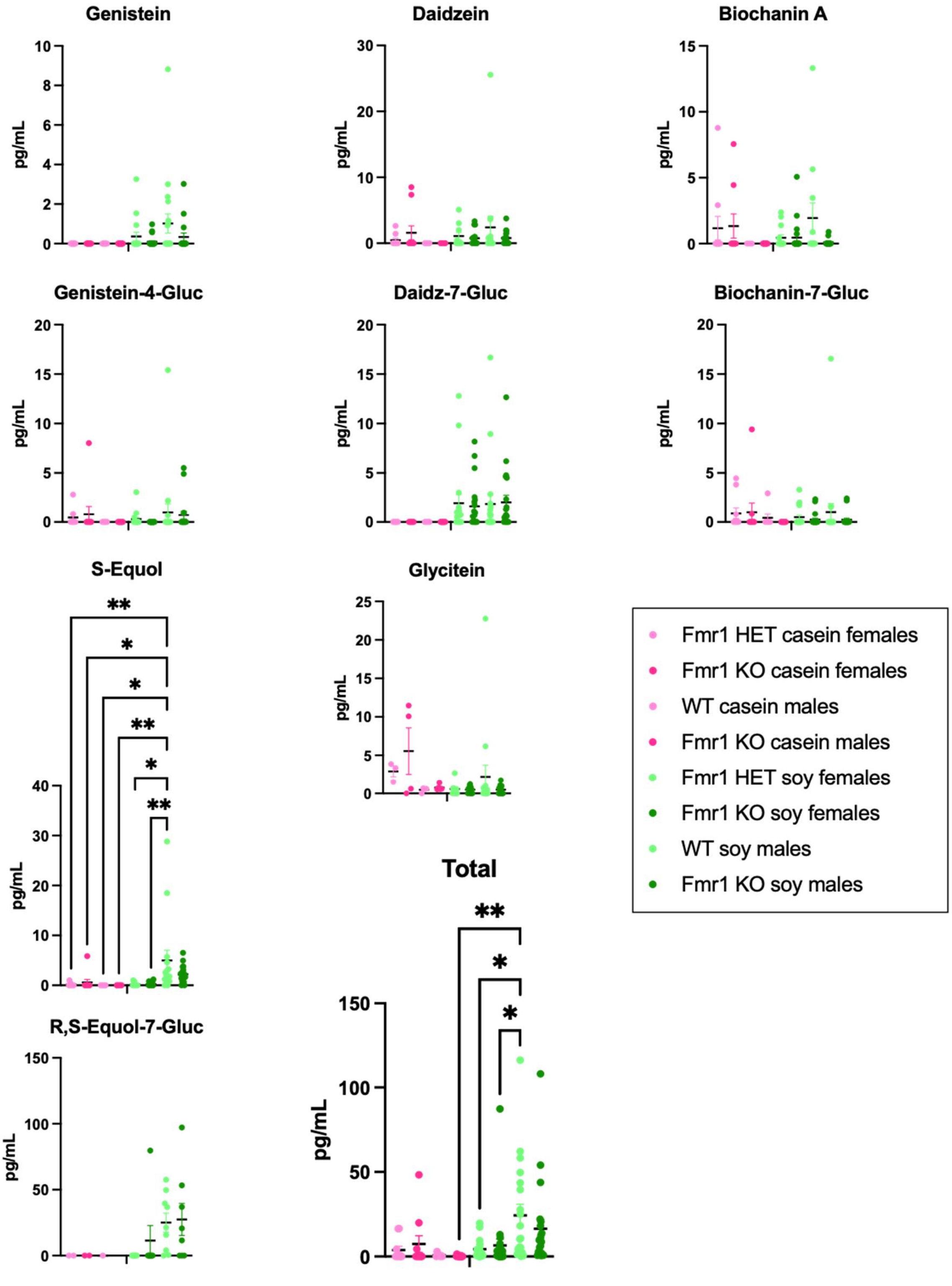

Figure S7. Blood-based phytoestrogen levels in response to sex, *Fmr1* genotype and soy protein isolate. Blood phytoestrogen levels were quantitated as a function of genotype and diet. Data were analyzed with an ANOVA 2-way model and Tukey's multiple comparison test. The data presented here are from blood samples collected at 8 months of age from the same mice that underwent behavioral and amino acid analyses; however, due to lockdown of the testing laboratory during COVID restrictions, there were problems with sample degradation during prolonged freezer storage.

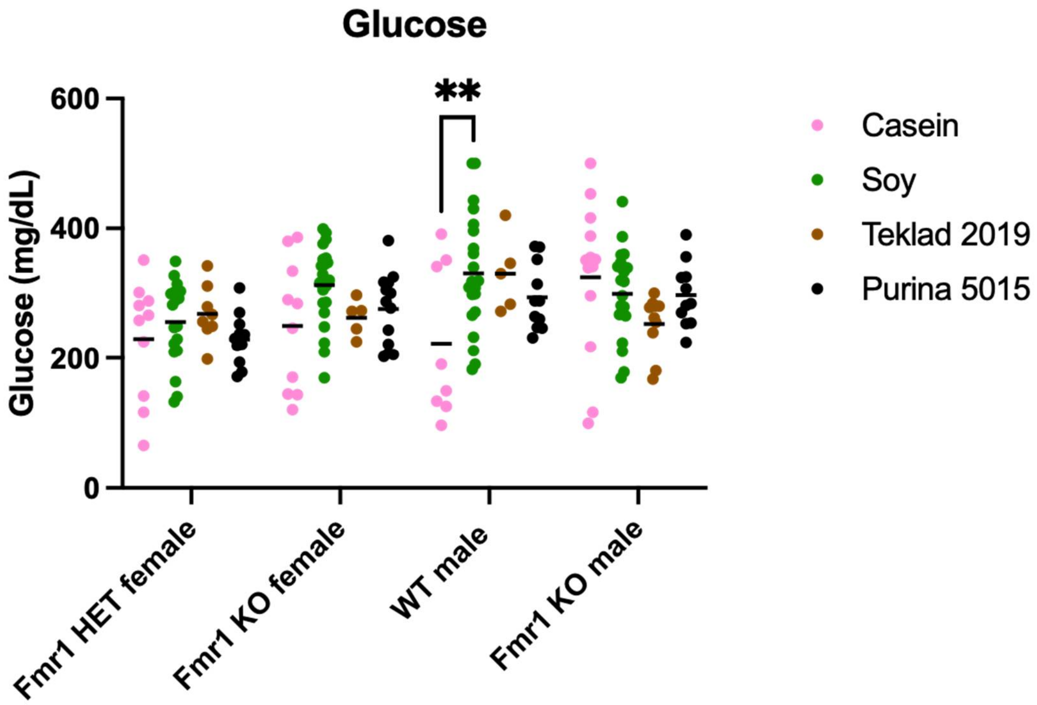

Figure S8. Glucose levels in response to sex, *Fmr1* genotype and soy protein isolate. Glucose levels were measured at euthanization in *Fmr1*^{HET} females fed casein diet (pink, n=10), soy diet (green, n=19), Teklad 2019 (brown, n=8) or Purina 50515 (black, n=11); *Fmr1*^{KO} females fed casein diet (pink, n=10), soy diet (green, n=22), Teklad 2019 (brown, n=5), or Purina 5015 (black, n=13); WT males fed casein diet (pink, n=8), soy diet (green, n=21), Teklad 2019 (brown, n=5), or Purina 5015 (black, n=11); and *Fmr1*^{KO} males fed casein diet (pink, n=13), soy diet (green, n=20), Teklad 2019 (brown, n=10), or Purina 5015 (black, n=11): Interaction F (9, 181) = 2.048, p=0.0365; Genotype F (3, 181) = 4.056, p=0.0081; Diet F (3, 181) = 3.316, p=0.0212.

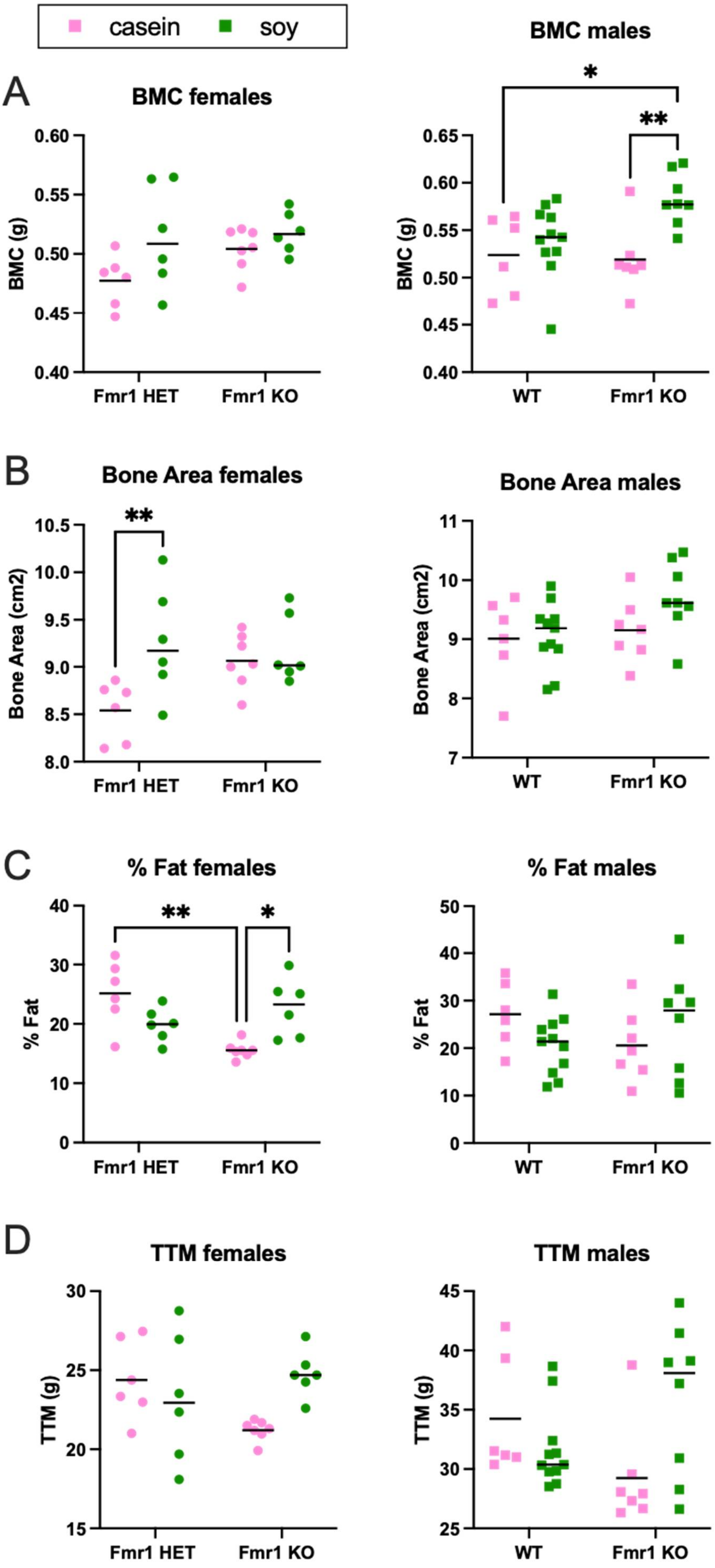

casein
soy
A
BMC females
BMC (g)
0.60
0.55
0.50
0.45
0.40
Fmr1 HET
Fmr1 KO
BMC males
*
**
0.65
0.60
0.55
0.50
0.45
0.40
WT
Fmr1 KO
B
Bone Area females
**
Bone Area (cm2)
10.5
10.0
9.5
9.0
8.5
8.0
Fmr1 HET
Fmr1 KO
Bone Area males
11
10
9
8
7
WT
Fmr1 KO
C
% Fat females
**
*
% Fat
40
30
20
10
0
Fmr1 HET
Fmr1 KO
% Fat males
50
40
30
20
10
0
WT
Fmr1 KO
D
TTM females
TTM (g)
30
25
20
15
Fmr1 HET
Fmr1 KO
TTM males
45
40
35
30
25
WT
Fmr1 KO

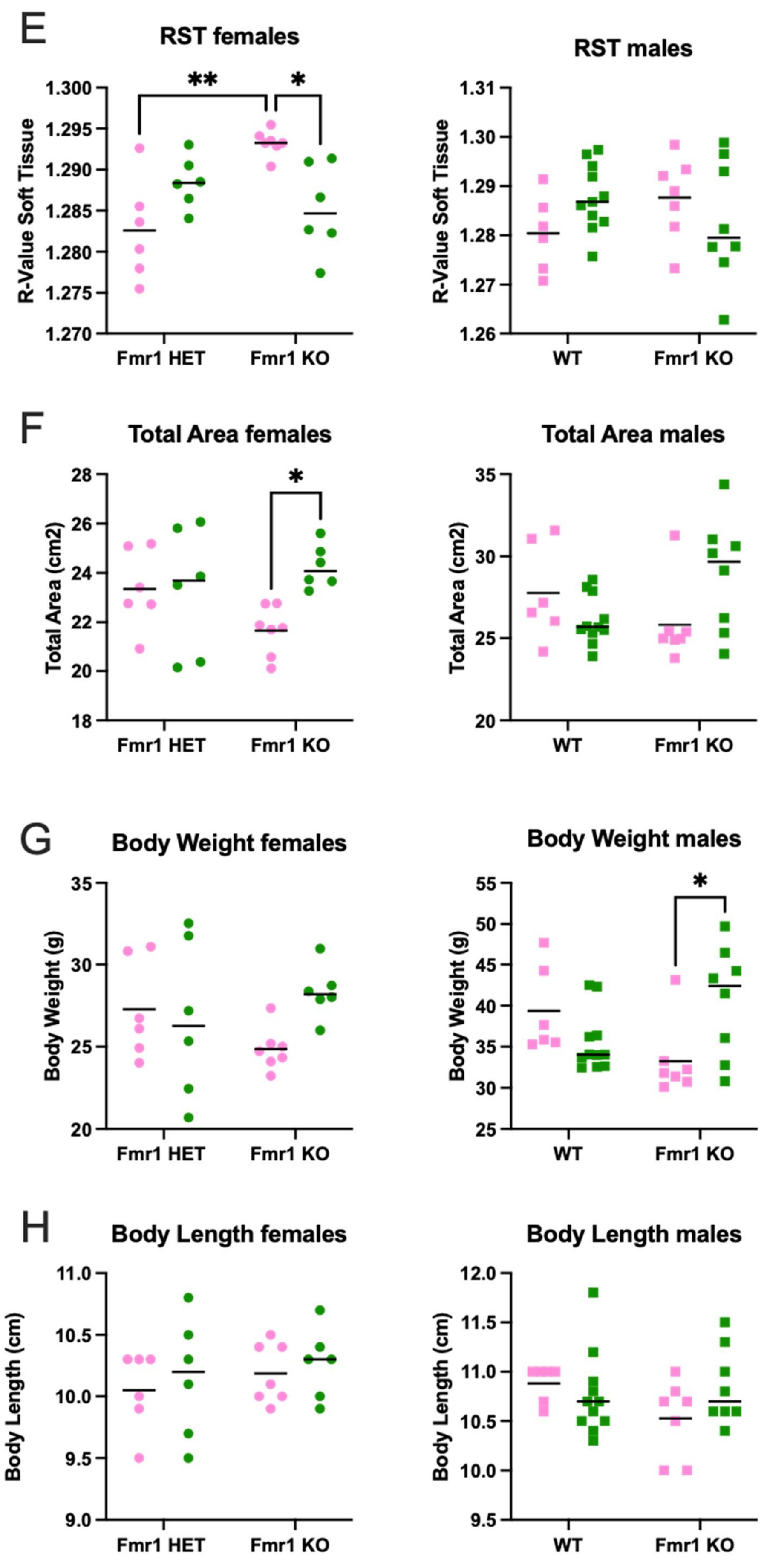
E
RST females
R-Value Soft Tissue
1.300
1.295
1.290
1.285
1.280
1.275
1.270
**
*
Fmr1 HET
Fmr1 KO
RST males
R-Value Soft Tissue
1.31
1.30
1.29
1.28
1.27
1.26
WT
Fmr1 KO
F
Total Area females
Total Area (cm2)
28
26
24
22
20
18
*
Fmr1 HET
Fmr1 KO
Total Area males
Total Area (cm2)
35
30
25
20
WT
Fmr1 KO
G
Body Weight females
Body Weight (g)
35
30
25
20
Fmr1 HET
Fmr1 KO
Body Weight males
Body Weight (g)
55
50
45
40
35
30
25
*
WT
Fmr1 KO
H
Body Length females
Body Length (cm)
11.0
10.5
10.0
9.5
9.0
Fmr1 HET
Fmr1 KO
Body Length males
Body Length (cm)
12.0
11.5
11.0
10.5
10.0
9.5
WT
Fmr1 KO

Figure S9. DEXA analysis in response to soy protein isolate in 8-month-old mice. Mice were scanned in a GE Lunar Piximus DEXA unit after euthanization at 8 months of age. *Fmr1*^{HET} (n=6 casein, n=6 soy) and *Fmr1*^{KO} (n=7 casein, n=6 soy) females (denoted by circles) and WT (n=6 casein, n=11 soy) and *Fmr1*^{KO} (n=7 casein, n=8 soy) males (squares) were analyzed separately with an ANOVA mixed-effects model and Tukey's multiple comparison test in response to casein (pink) and soy (green) diets. Variables not reported in Figure 9 are reported here: (A) BMC females: Interaction $F(1, 21) = 1.122$, $p=0.3015$; Genotype $F(1, 21) = 1.199$, $p=0.1721$; Diet $F(1, 21) = 5.520$, $p=0.0287$. BMC males: Interaction $F(1, 28) = 3.474$, $p=0.0729$; Genotype $F(1, 28) = 2.242$, $p=0.1455$; Diet $F(1, 28) = 9.312$, $p=0.0049$. (B) Bone area females: Interaction $F(1, 21) = 3.512$, $p=0.0749$; Genotype $F(1, 21) = 2.000$, $p=0.1720$; Diet $F(1, 21) = 7.033$, $p=0.0149$. Bone area males: Interaction $F(1, 28) = 1.330$, $p=0.2585$; Genotype $F(1, 28) = 3.294$, $p=0.0803$; Diet $F(1, 28) = 2.057$, $p=0.1626$. (C) % Fat females: Interaction $F(1, 21) = 15.90$, $p=0.0007$; Genotype $F(1, 21) = 4.497$, $p=0.0460$; Diet $F(1, 21) = 0.3808$, $p=0.5438$. % Fat males: Interaction $F(1, 28) = 3.569$, $p=0.0693$; Genotype $F(1, 28) = 0.1382$, $p=0.7129$; Diet $F(1, 28) = 0.1377$, $p=0.7134$. (D) TTM females: Interaction $F(1, 21) = 5.641$, $p=0.0272$; Genotype $F(1, 21) = 0.6681$, $p=0.4229$; Diet $F(1, 21) = 1.480$, $p=0.2373$. TTM males: Interaction $F(1, 28) = 6.941$, $p=0.0136$; Genotype $F(1, 28) = 0.06310$, $p=0.8035$; Diet $F(1, 28) = 1.360$, $p=0.2535$. (E) RST females: Interaction $F(1, 21) = 15.91$, $p=0.0007$; Genotype $F(1, 21) = 4.502$, $p=0.0459$; Diet $F(1, 21) = 0.3820$, $p=0.5432$. RST males: Interaction $F(1, 28) = 3.570$, $p=0.0692$; Genotype $F(1, 28) = 0.1383$, $p=0.7127$; Diet $F(1, 28) = 0.1380$, $p=0.7131$. (F) Total area females: Interaction $F(1, 21) = 4.143$, $p=0.0546$; Genotype $F(1, 21) = 0.3176$, $p=0.5790$; Diet $F(1, 21) = 3.827$, $p=0.0638$. Total area males: Interaction $F(1, 28) = 6.475$, $p=0.0167$; Genotype $F(1, 28) = 0.1893$, $p=0.6668$; Diet $F(1, 28) = 0.5552$, $p=0.4624$. (G) Body weight (weighed on a balance prior to DEXA) females: Interaction $F(1, 21) = 2.988$, $p=0.0985$; Genotype $F(1, 21) = 0.1051$, $p=0.7490$; Diet $F(1, 21) = 1.460$, $p=0.2403$. Body weight males: Interaction $F(1, 28) = 9.453$, $p=0.0047$; Genotype $F(1, 28) = 0.08602$, $p=0.7715$; Diet $F(1, 28) = 0.9257$, $p=0.3442$. (H) body length (measured prior to DEXA) females: Interaction $F(1, 21) = 0.004795$, $p=0.9455$; Genotype $F(1, 21) = 0.8418$, $p=0.3693$; Diet $F(1, 21) = 0.4327$, $p=0.5178$. Body length males: Interaction $F(1, 28) = 2.640$, $p=0.1154$; Genotype $F(1, 28) = 0.9773$, $p=0.3313$; Diet $F(1, 28) = 0.5521$, $p=0.4637$.

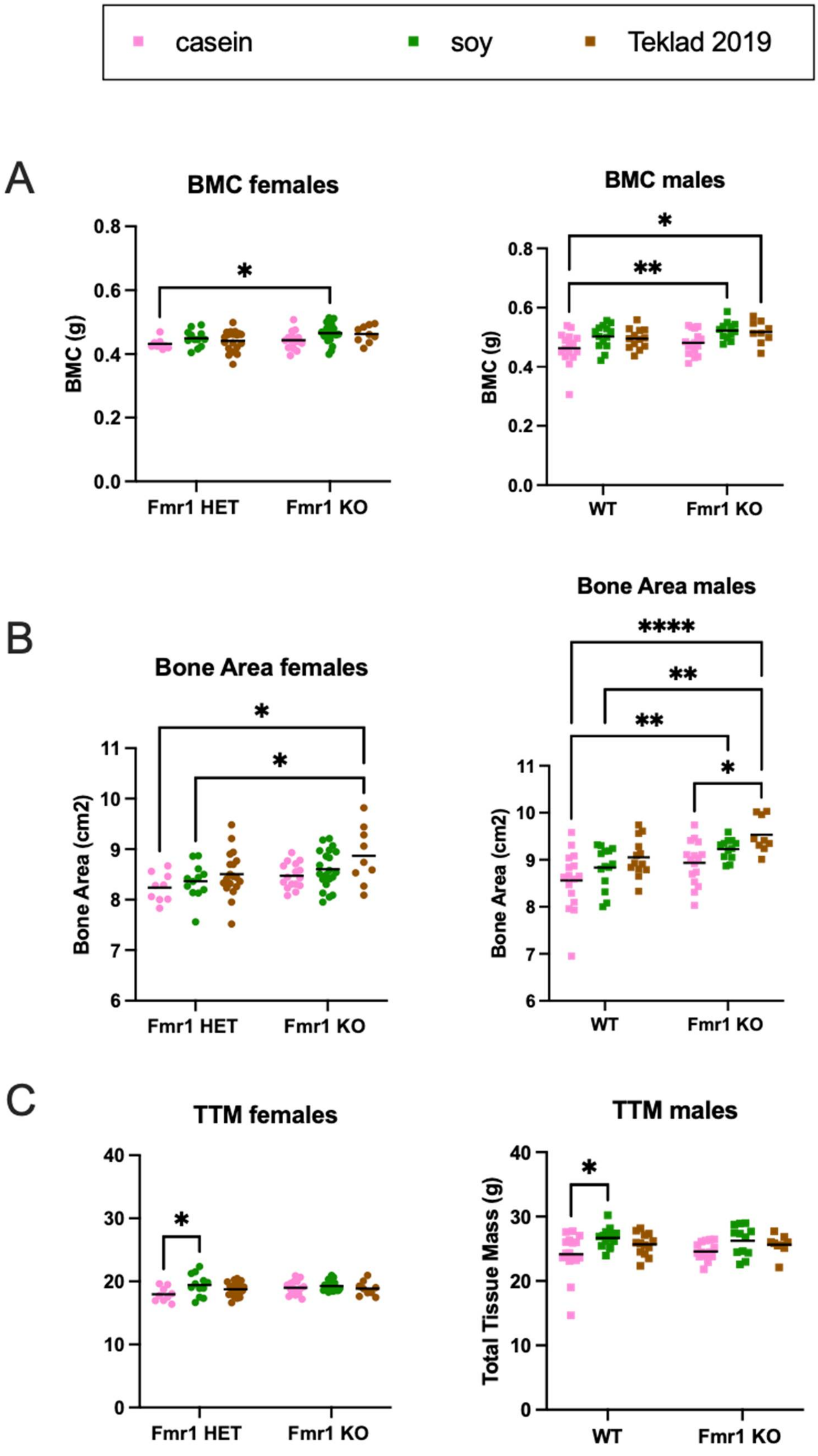

casein
soy
Teklad 2019
A
BMC females
BMC (g)
Fmr1 HET
Fmr1 KO
BMC males
WT
Fmr1 KO
B
Bone Area females
Bone Area (cm2)
Bone Area males
C
TTM females
TTM males
Total Tissue Mass (g)

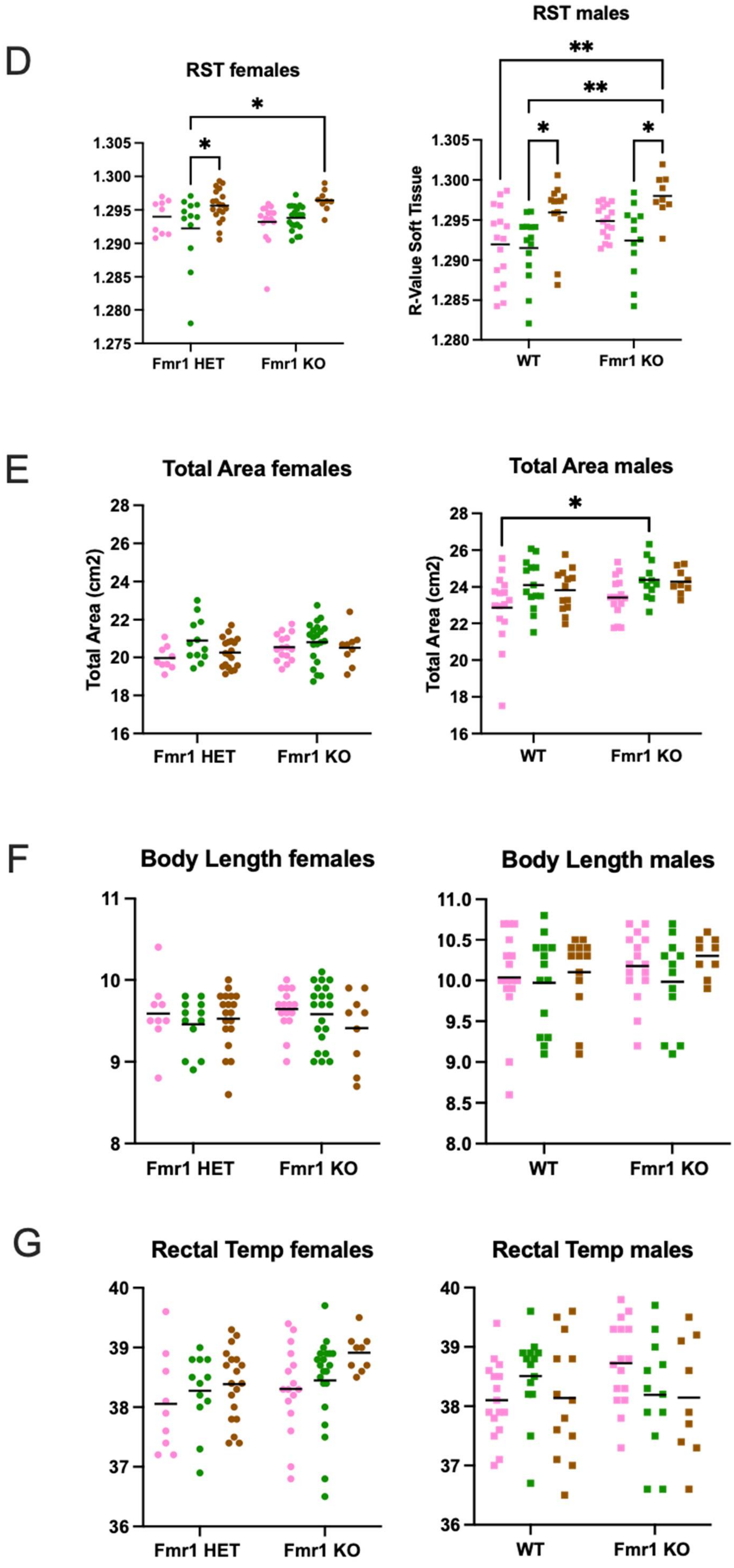
D
RST females
RST males
R-Value Soft Tissue
1.305
1.300
1.295
1.290
1.285
1.280
1.275
*
**
Fmr1 HET
Fmr1 KO
WT
E
Total Area females
Total Area males
Total Area (cm2)
28
26
24
22
20
18
16
F
Body Length females
Body Length males
11
10
9
8
11.0
10.5
10.0
9.5
9.0
8.5
8.0
G
Rectal Temp females
Rectal Temp males
40
39
38
37
36

Figure S10. DEXA analysis in response to soy protein isolate in 4-month-old mice. Mice were scanned in a GE Lunar Piximus DEXA unit after euthanization at 4 months of age. *Fmr1*^{HET} (n=9 casein, n=12 soy, n=19 Teklad 2019) and *Fmr1*^{KO} (n=16 casein, n=21 soy, n=9 Teklad 2019) females (circles) and WT (n=16 casein, n=14 soy, n=13 Teklad 2019) and *Fmr1*^{KO} (n=16 casein, n=12 soy, n=9 Teklad 2019) males (squares) were analyzed separately with an ANOVA mixed-effects model and Tukey's multiple comparison test in response to casein (pink), soy (green) and Teklad 2019 (brown) diets. (A) BMC females: Interaction F (2, 80) = 0.1721, *p*=0.8422; Genotype F (2, 80) = 3.232, *p*=0.0447; Diet F (1, 80) = 6.429, *p*=0.0132. BMC males: Interaction F (2, 74) = 0.02042, *p*=0.9798; Genotype F (2, 74) = 7.857, *p*=0.0008; Diet F (1, 74) = 4.424, *p*=0.0388. (B) Bone area females: Interaction F (2, 80) = 0.2193, *p*=0.8035; Genotype F (2, 80) = 4.455, *p*=0.0146; Diet F (1, 80) = 9.870, *p*=0.0024. Bone area males: Interaction F (2, 74) = 0.08824, *p*=0.9156; Genotype F (2, 74) = 9.273, *p*=0.0003; Diet F (1, 74) = 16.07, *p*=0.0001. (C) TTM females: Interaction F (2, 80) = 0.8430, *p*=0.4342; Genotype F (2, 80) = 3.161, *p*=0.0477; Diet F (1, 80) = 1.381, *p*=0.2434. TTM males: Interaction F (2, 74) = 0.07698, *p*=0.9260; Genotype F (2, 74) = 5.677, *p*=0.0051; Diet F (1, 74) = 2.045, *p*=0.1569. (D) RST females: Interaction F (2, 80) = 1.028, *p*=0.3623; Genotype F (2, 80) = 7.338, *p*=0.0012; Diet F (1, 80) = 0.5924, *p*=0.4438. RST males: Interaction F (2, 74) = 0.4755, *p*=0.6234; Genotype F (2, 74) = 10.27, *p*=0.0001; Diet F (1, 74) = 5.070, *p*=0.0273. (E) TTM females: Interaction F (2, 80) = 0.8430, *p*=0.4342; Genotype F (2, 80) = 3.161, *p*=0.0477; Diet F (1, 80) = 1.381, *p*=0.2434. TTM males: Interaction F (2, 74) = 0.07698, *p*=0.9260; Genotype F (2, 74) = 5.677, *p*=0.0051; Diet F (1, 74) = 2.045, *p*=0.1569. (F) Body length females: Interaction F (2, 80) = 0.7684, *p*=0.4672; Genotype F (2, 80) = 1.035, *p*=0.3601; Diet F (1, 80) = 0.06435, *p*=0.8004. Body length males: Interaction F (2, 74) = 0.2257, *p*=0.7985; Genotype F (2, 74) = 1.206, *p*=0.3052; Diet F (1, 74) = 1.060, *p*=0.3065. (G) Rectal temperature females: Interaction F (2, 80) = 0.4878, *p*=0.6158; Genotype F (2, 80) = 2.863, *p*=0.0630; Diet F (1, 80) = 4.166, *p*=0.0445. Rectal temperature males: Interaction F (2, 74) = 2.418, *p*=0.0961; Genotype F (2, 74) = 0.7107, *p*=0.4946; Diet F (1, 74) = 0.3105 *p*=0.5791.

Table S1. Casein and soy protein-based matched diets.

Ingredient	Envigo TD.180374 (casein-based); g/kg	Envigo TD.180375 (soy-based); g/kg
casein	200.0	0.0
isolated soy protein	0.0	200.0
L-cystine	3.0	1.3
L-methionine	0.0	2.4
corn starch	394.886	405.726
maltodextrin	132.0	132.0
sucrose	100.0	100.0
soybean oil	70.0	70.0
cellulose	50.0	50.0
mineral mix, AIN-93G-MX (94046)	35.0	0.0
trace mineral mix, AIN-93G (06095)	0.0	5.0
sodium chloride	2.5	0.0
calcium phosphate, dibasic	0.0	9.5
calcium carbonate	0.0	4.5
potassium chloride	0.0	6.3
magnesium oxide	0.0	0.66
vitamin mix, AIN-93G-VX (94047)	10.0	10.0
chlorine bitartrate	2.5	2.5
TBHQ, antioxidant	0.014	0.014
food color	0.1 (red)	0.1 (green)

Table S2. Phytoestrogen mass spectrometry conditions.[1]

Compound	Parent Ion	Product Ions	Period	Instrument Conditions
Daid-7-Gluc	429	253, 113, 175	0-1.9 min	CuG: 38.0, CoG: Med, ISV: -3000V, Temp: 550 °C, SG1: 35,SG2: 33.0, DP: -40V
Gen-4-Gluc	445	113, 269, 175	1.9-2.3 min	CuG: 38.0, CoG: Med, ISV: -2500V, Temp: 525 °C, SG1: 35, SG2: 33.0, DP: -100V
Eq-7-Gluc	417	113, 241, 175	1.9-2.3 min	CuG: 38.0, CoG: Med, ISV: -2500V, Temp: 525 °C, SG1: 35, SG2: 33.0, DP: -55V
Bio-A-Gluc	459	283, 175, 113	2.3-2.55 min	CuG: 30.0, CoG: Med, ISV: -2000V, Temp: 550 °C, SG1: 20, SG2: 35.0, DP: -90V
Daidzein	253	208, 223, 132	2.55-3.15 min	CuG: 33.0, CoG: Med, ISV: -1600V, Temp: 470 °C, SG1: 30, SG2: 40.0, DP: -130V
Glycitein	283	240, 184, 268	2.55-3.15 min	CuG: 33.0, CoG: Med, ISV: -1600V, Temp: 470 °C, SG1: 30, SG2: 40.0, DP: -75V
Genistein	269	133, 159, 181	3.15-3.60 min	CuG: 32.0, CoG: Med, ISV: -2300V, Temp: 450 °C, SG1: 33, SG2: 35.0, DP: -155V
Equol	241	121, 135, 147	3.15-3.60 min	CuG: 32.0, CoG: Med, ISV: -2300V, Temp: 450 °C, SG1: 33, SG2: 35.0, DP: -110V
Chrysin	253	143, 107, 209	3.60-4.50 min	CuG: 33.0, CoG: Med, ISV: -1600V, Temp: 475 °C, SG1: 35, SG2: 33.0, DP: -110V
Biochanin A	283	239, 211, 268	3.60-4.50 min	CuG: 33.0, CoG: Med, ISV: -1600V, Temp: 475 °C, SG1: 35, SG2: 33.0, DP: -105V

[1]Isoflavone compounds quantitated by LC/MS/MS showing parent ion, product ions, period time and optimized instrument parameters. Abbreviations: CuG-Curtain Gas; CoG-Collision Gas; ISV-Ion Source Voltage; Temp-Source temperature; SG1-Source Gas1; SG2-Source Gas 2; DP-Declustering Potential.

Table S3. Abdominal disease [1] as a function of genotype and diet.

Genotype / Diet	Casein (%, N)		Soy (%, N)		Teklad 2019 (%, N)		Purina 5015 (%, N)	
Fmr1^{HET} female	30%	10	16% [5]	19	0%	8	0%	11
Fmr1^{KO} female	40% [3]	10	0%	22	0%	5	7.7%	13
WT male	25%	8	4.8%	21	0%	5	0%	11
Fmr1^{KO} male [2]	31% [4]	13	5%	20	20%	10	0%	11

[1] at euthanization, presence of diseased liver, enlarged spleen and/or enlarged gall bladder

[2] one mouse was excluded from the study due to malocclusion; also had penile protrusion

[3] Fisher exact test comparing casein and soy, p=0.0058

[4] Fisher exact test comparing casein and soy, p=0.066

[5] Fisher exact test comparing *Fmr1*^{HET} and *Fmr1*^{KO} females, p=0.091

Table S4. Diseased liver as a function of genotype and diet.

Genotype / Diet	Casein (%, N)		Soy (%, N)		Teklad 2019 (%, N)		Purina 5015 (%, N)	
Fmr1^{HET} female	30%	10	0%	19	0%	8	0%	11
Fmr1^{KO} female	40%	10	0%	22	0%	5	7.7%	13
WT male	25%	8	0%	21	0%	5	0%	11
Fmr1^{KO} male	31%	13	0%	20	20%	10	0%	11

Table S5. Abdominal disease as a function of diet.

Genotype / Diet	%	N	Fisher Exact Test (versus casein)
Casein	32%	41	---
Soy	6.1%	82	p=0.0003
Teklad 2019	7.1%	28	p=0.018
Purina 5015	2.2%	46	p=0.0002
